# Oral Potentially Malignant Disorders: Etiology, Pathogenesis, and Transformation Into Oral Cancer

**DOI:** 10.3389/fphar.2022.825266

**Published:** 2022-04-20

**Authors:** Pratima Kumari, Priyanka Debta, Anshuman Dixit

**Affiliations:** ^1^ Computational Biology and Bioinformatics Lab, Institute of Life Sciences, Bhubaneswar, India; ^2^ Regional Centre for Biotechnology (RCB), Faridabad, India; ^3^ Department of Oral Pathology and Microbiology, Institute of Dental Sciences, Siksha “O” Anusandhan Deemed to be University, Bhubaneswar, India

**Keywords:** malignant transformation, oral lichenoid lesions, proliferative verrucous leukoplakia, oral lichen planus, oral submucous fibrosis, erythroplakia, leukoplakia, oral potentially malignant disorders

## Abstract

Among oral diseases, oral cancer is a critical health issue due to its life-threatening potential. Globocan, in its 2020 report, estimated ∼0.37 million new cases of oral cancer, with the majority of them coming from the Asian continent. The WHO has anticipated a rise in the incidences of oral cancer in the coming decades. Various factors, such as genetic, epigenetic, microbial, habitual, and lifestyle factors, are closely associated with oral cancer occurrence and progression. Oral lesions, inherited genetic mutations (dyskeratosis congenital syndrome), and viral infections (HPV) are early signs of oral cancer. Lesions with dysplastic features have been categorized under oral potentially malignant disorders (OPMDs), such as oral leukoplakia, erythroplakia, oral submucous fibrosis (OSMF), and proliferative verrucous leukoplakia, are assumed to have a high risk of malignancy. The incidence and prevalence of OPMDs are recorded as being high in South-Asian countries. Early detection, prevention, and treatment of OPMDs are needed to prevent its malignant transformation into oral cancer. Many advanced diagnostic techniques are used to predict their progression and to assess the risk of malignant transformation. This communication provides insight into the importance of early detection and prevention of OPMDs.

## Graphical Abstract

**(A)** A representative diagram of changes occurring in buccal mucosa due to exposure to various carcinogenic factors. The red arrow shows the direction of changes associated with the progression of oral cancer. **(B)** The circular map shows the chromosomes involve in genetic alternation. 
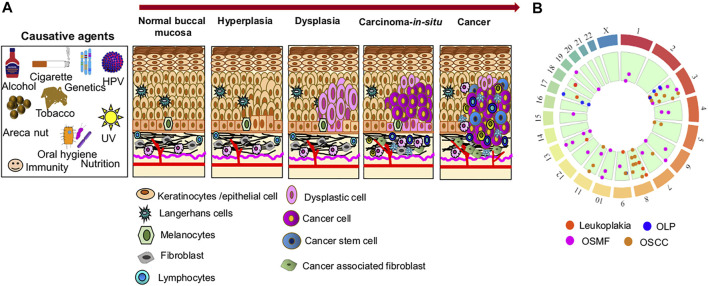

## 1 Introduction

The Globocan 2020 report addresses the overall burden of cancer incidence and mortality as a cause of premature death. About 0.37 million new cases and 0.17 million new deaths were recorded in the case of lip and oral cavity cancer. A higher burden of lip and oral cavity cancer was reported (10.2 per 100,000) in countries that scored lower on the Human Development Index (HDI) such as India (Sung et al., 2021).

**FIGURE 1:**
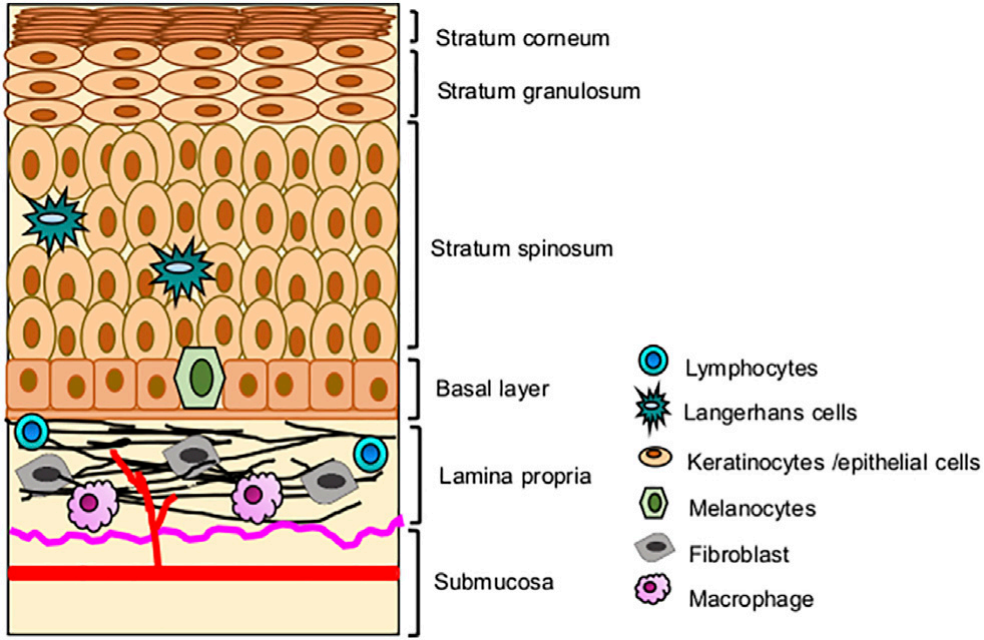
Systematic representation of normal buccal mucosa.

Oral cancer, a disease of epithelial origin, is associated with multifactorial etiology, such as genetic, epigenetic, habitual (tobacco/areca nut/cigarette/alcohol), and microbial factors, which often vary with geographical regions or ethnic groups. A series of histopathological changes have been documented in normal mucosa during oral cancer progression. These changes include hyperplasia, dysplasia, carcinoma *in situ*, and, finally, oral cancer (Al-Swiahb et al., 2010; Banerjee and Chatterjee, 2015). The primary determinants of oral cancer progression are habitual factors, as changing underlying habits can revert or slow the cancer progression mainly at the benign/dysplastic stage (Hunter and Yeoman, 2013). More often, the appearance of abnormalities in the oral mucosa (atypia) may be the beginning of pre-cancerous conditions (Figure 1) (Rajendran, 2004). In comparison, oral epithelial dysplasia (OED) is marked as an intermediate stage determining the malignancy potential of varied premalignant precursors in oral cancer development. Thus, OED is used as an indication for malignant transformation (MT) of oral potentially malignant disorders (OPMDs) (Warnakulasuriya et al., 2007; Thomas et al., 2012). The development of oral cancer from OPMDs is common, especially in South Asian countries like India where tobacco and areca nut consumption is prevalent (Kramer et al., 1978).

The available literature has been focused primarily on the identification of these OPMDs. Differences in the oral manifestation of these lesions have always been a big hurdle in clinical decision making. Clinical and histological parameters have been revised many times by the WHO to reduce the ambiguity in uniform reporting of OPMDs.

Information regarding OPMDs has been scattered in the literature with few attempts to group it together having been made in the past. Therefore, it is pertinent to compile the available information in a structured manner regarding the diseases process, characteristics, and diagnosis of OPMDs that underlie the majority of oral cancer cases. Needless to say, it is important to have a better understanding of the current state of the art in diagnosis, prognosis, and therapeutics aspects of OPMDs to develop better strategies and the current article is an attempt in this direction.

## 2 Oral Potentially Malignant Disorders

European physicians originally introduced OPMDs in 1805 as “pre-cancer,” suggesting that oral pre-cancers are benign conditions that may eventually develop into invasive malignancies in the long run (Ray, 2017). Thereafter, constant efforts were made to characterize and define the term oral pre-cancerous conditions, and in 1978 the WHO proposed the terms “pre-cancerous conditions” and “pre-cancerous lesion.” Later, the WHO merged the term lesions and condition to represent all the clinical manifestations that carry a risk to oral cancer as “oral potentially malignant disorders” (OPMDs) (Table 1) (Van der Waal, 2019). OPMDs are defined as “any oral mucosal abnormality that is associated with a statistically increased risk of developing oral cancer” (Warnakulasuriya et al., 2020a). Characteristically, OPMDs present with diverse clinical attributes, such as color variations (white, red, and mixed white-red), morphological changes (plaque/plateau, smooth, grooved, wrinkled, granular, atrophic), and different sizes, involving different anatomical sites in the oral cavity (Williams et al., 2008; Farah et al., 2014).

**TABLE 1:** Definition of terms used for characterization of oral pre-cancer disorders.

Terms	Definition
Oral precancerous lesion	A morphologically altered tissue that has greater potential to cause malignancy than the corresponding normal counterpart does George et al. (2011), Sarode et al. (2014)
Oral precancerous conditions	A generalized condition that does not necessarily show clinical alteration of oral mucosa but is associated with a significantly increased risk of cancer development George et al. (2011), Sarode et al. (2014)
OPMDs	Any clinical presentations that carry a high risk of cancer development in the oral cavity, whether in a clinically definable precursor lesion or in clinically normal mucosa Warnakulasuriya et al. (2020b)

Interestingly, not all oral lesions develop into oral cancer, and some oral cancers develop from non-dysplastic lesions. Some morphological alterations are highly susceptible to malignant transformation; for example, in leukoplakia patients, malignancy may arise elsewhere in clinically normal mucosa (Speight, 2007).

Epidemiological studies have shown that 4.47% of the world’s population may have OPMDs. Among them, the majority of the cases are reported in the South Asian population (Mello et al., 2018). Men are a frequently affected group, presumably due to a higher prevalence of tobacco and alcohol use among them compared to women. Various studies have estimated that the prevalence of OPMDs varies among the populations and is mainly associated with habits. The majority (∼80%) of oral cancer cases originate from OPMDs (Kramer et al., 1978; Gupta et al., 1989). The overall malignant transformation (MT) rate of OPMDs is 7.9%, indicating the seriousness of the problem (Iocca et al., 2020).

**FIGURE 2:**
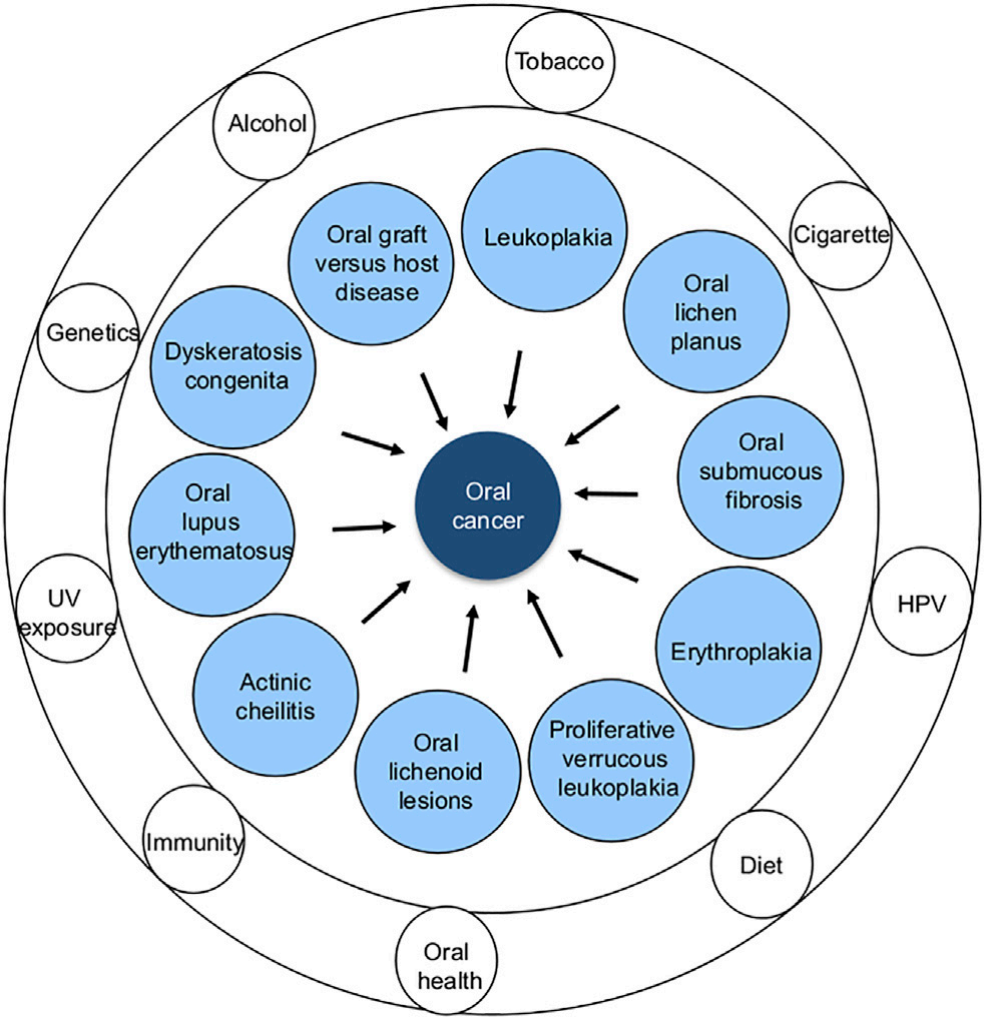
Different types of OPMDs (inner circle) and common risk factors (outer rings) associated with oral cancer development.

Currently identified OPMDs that are associated with a high risk of MT include leukoplakia, proliferative verrucous leukoplakia (PVL), erythroplakia, oral lichen planus (OLP), oral submucous fibrosis (OSMF), actinic cheilitis, palatal lesions of reverse cigar smoking, discoid lupus erythematosus, dyskeratosis congenita, oral lichenoid lesions, and oral graft versus host disease (Figure 2) (Mortazavi et al., 2014; Warnakulasuriya et al., 2020a).

### 2.1 Leukoplakia

#### 2.1.1 Definition

In 2005, a new definition of leukoplakia was proposed: “A white plaque of questionable risk having excluded (other) known diseases or disorders that carry no increased risk for cancer” (Warnakulasuriya et al., 2007). Various attempts have been made to classify leukoplakia as the presence of other oral white lesions has complicated the successful identification of leukoplakia and its classification, resulting in confusion and indecision in uniform reporting of this lesion (Lodi and Porter, 2008). Shanbhag et al. have suggested the use of the term “white lesion” instead of “white plaque” as other oral lesions may interfere with the diagnosis of leukoplakia due to shared etiological and clinical features. They emphasized the identification of lesions (scrapable or non-scrapable lesions, reversible or irreversible), and their relationship with common etiologic factors (tobacco, alcohol, betel quid) is important. Thus, they proposed a new definition for leukoplakia: “a predominantly white, irreversible, non-scrapable lesion of the oral mucosa that cannot be characterized clinically or histopathologically as any other lesion/disease and has increased risk of cancer occurrence than its normal counterpart and is usually associated with consumption of tobacco, betel quid, and alcohol, but otherwise can be of idiopathic in nature” (Shanbhag, 2017).

#### 2.1.2 Epidemiology and Etiology

The current global prevalence of leukoplakia is 4.11%, with the highest occurrence being in the Asian population (7.77%) (Mello et al., 2018). The definitive cause of leukoplakia is unclear. However, the most common risk factors involve the use of tobacco either in smoke (mainly) or smokeless form together with chronic alcohol consumption (Bánóczy et al., 2001; Sabashvili et al., 2018). It is necessary to distinguish causative factors to rule out other white oral lesions such as stomatitis nicotina, the most common lesions in smokers (Schwartz, 1965). The possible implication of viruses like human papillomavirus (HPV) and Epstein Barr virus (EBV) in the manifestation of leukoplakia remains unclear (Bhargava et al., 2016; Khammissa et al., 2016).

#### 2.1.3 Pathogenesis

An understanding of the molecular pathogenesis of leukoplakia is important to minimize the chances of oncogenic transformation. Various studies have reported the presence of molecular abnormalities associated with both leukoplakia and oral cancer (Henrichsen et al., 2009). Alterations (insertions/deletions/mutations) in the chromosomal region(s) with tumor suppressor genes or proto-oncogenes increase the carcinogenic potential of OPMDs. Chromosomal deletion in 3p14 and 9p21 region in leukoplakia likely increases the invasive potential of oral cancer (Mao et al., 1996). Deletion in 4q, 8p, 11q, and 17p region are also seen in leukoplakia (Rosin et al., 2000; Zhang et al., 2001). Allelic imbalances in the 3p21, 8p21-23, 9p21, 13q14.2, 17p13.1, and 18q21.1 regions plausibly help in oral cancer progression (Partridge et al., 1999). Multiple copy number variations (CNVs) at 3p, 8p, 9p, 11q, 13q, 18q, and 17p regions are considered genetic markers for the progressive type of epithelial dysplasia (Partridge et al., 1999; Mithani et al., 2007; Kil et al., 2016). The DNA ploidy level serves as an important determinant of genetic stability and alteration in genomic sequence. A considerable ploidy level has been observed in OPMDs and subsequently in oral cancer. Khanna et al. (2008) showed in as tudy that the fraction of the DNA ploidy level in leukoplakia is markedly high in comparison to normal mucosa. The aneuploidy level in oral cancer and leukoplakia was found to be 64 and 20% respectively, while normal mucosa biopsies were reported to be diploid. Similarly, leukoplakia with dysplasia has an aneuploidy rate of 38% while leukoplakia without dysplasia has a 14% aneuploidy level (Khanna et al., 2010). A study conducted on genomic stability in the Indian population showed that aneuploidy levels in oral cancer and leukoplakia were 79.1% and 32.4% respectively. High-grade dysplastic cases of ulcerated, nodular, and verrucous leukoplakia have shown aneuploidy levels of 13.1, 22.2, and 28% respectively, whereas the diploid genome as reported in the majority (85%) of the non-dysplastic or mild dysplastic cases (Thomas et al., 2020). Thus, it is evident that genomic content changes drastically during MT of leukoplakia, and monitoring these changes can provide important information for diagnosis and potential stratification of patients. Besides genomic changes, abnormal protein-related behaviors were also reported in OPMDs. The immortality of cancer cells has frequently been related to telomerase activity. Overexpression of telomerase enzyme maintains the structure and function of the telomeres and prevents apoptosis of cancerous cells (Mishra et al., 2019). In total, 60% of the leukoplakia cases were shown to have positive telomerase activity and were well correlated with the clinical type and histologic grade in leukoplakia. Other clinical types of leukoplakia were also reported to show positive telomerase activity with a rate of 31% in moderate to severe dysplasia, 44% in ulcerated and nodular, and 36% in verrucous leukoplakia (Thomas et al., 2020). Another tumor marker, p53, was reported upregulated in leukoplakia, and approximately 1.6% of leukoplakia cases were p53 positive. Moreover, oral cancer developed from leukoplakia showed an increased p53 positive status with a rate of 3.4% (Cui et al., 2013). The p53 expression rate and intensity were reported to be higher in the high-grade group in comparison to the low-grade group with a higher lymph node metastatic potential than p53 negative cancer (Cui et al., 2013; Okoturo et al., 2018). Other molecular markers also, at the tissue level, showed alternation as atypical (hyperplastic and dysplastic) conditions arose in the oral epithelium. These markers include keratins, integrin, TGF-α, Cyclin D1, MMP1, and MMP9 (Martorell-Calatayud et al., 2009). For example, keratin (K5/K14) commonly expressed in the normal epithelium is found to be expressed in parabasal and stratum spinosum indicating hyperplasia of basal cells. In contrast, hyperplasia results in the increased level of K4/K13 and K1/K10 in normal mucosa (Williams et al., 1991; Bloor et al., 2001). Integrin α_v_β_6_, which is absent in the normal epithelium, is found to be expressed in dysplastic leukoplakia and significantly increases with oral cancer progression with a frequency of 27%–90% (Hamidi et al., 2000). The existence of an immunosuppressive tumor microenvironment, inhibiting the action of infiltrating immune cells, and the presence of immune checkpoints, including PD1/PD-L1 (programmed death/programmed death–ligand 1), all together contribute to cancer immune escape and aid cancer progression. Reports have shown that the presence of CD8^+^ and CD163 + cells (macrophages) was important for immune response. However, increased expression of PD-L1 in both dysplastic epithelial cells and subepithelial infiltrating cells (macrophages) was seen in leukoplakia, indicating the repression of anti-tumor immunity. Increased expression of PD-L1 and its positive correlation with CD163 + cells infiltration was reported, whereas the expression of PD-L1+ in sub epithelial cells was inversely related with CD8^+^ cell infiltration. Furthermore, the PD-1/PD-L1 pathway also influenced the early development of oral cancer. Thus, the blockade of PD-L1 can increase the CD8^+^ cell infiltration into the tumor environment to promote an anti-tumor immune response (Miura et al., 2017; Stasikowska‐Kanicka et al., 2018; Massi et al., 2019). Although genomic- and protein-based markers have been identified in leukoplakia, further investigation are necessary to determine the diagnostic and prognostic value of the markers.

#### 2.1.4 Clinical and Histological Presentations

**FIGURE 3:**
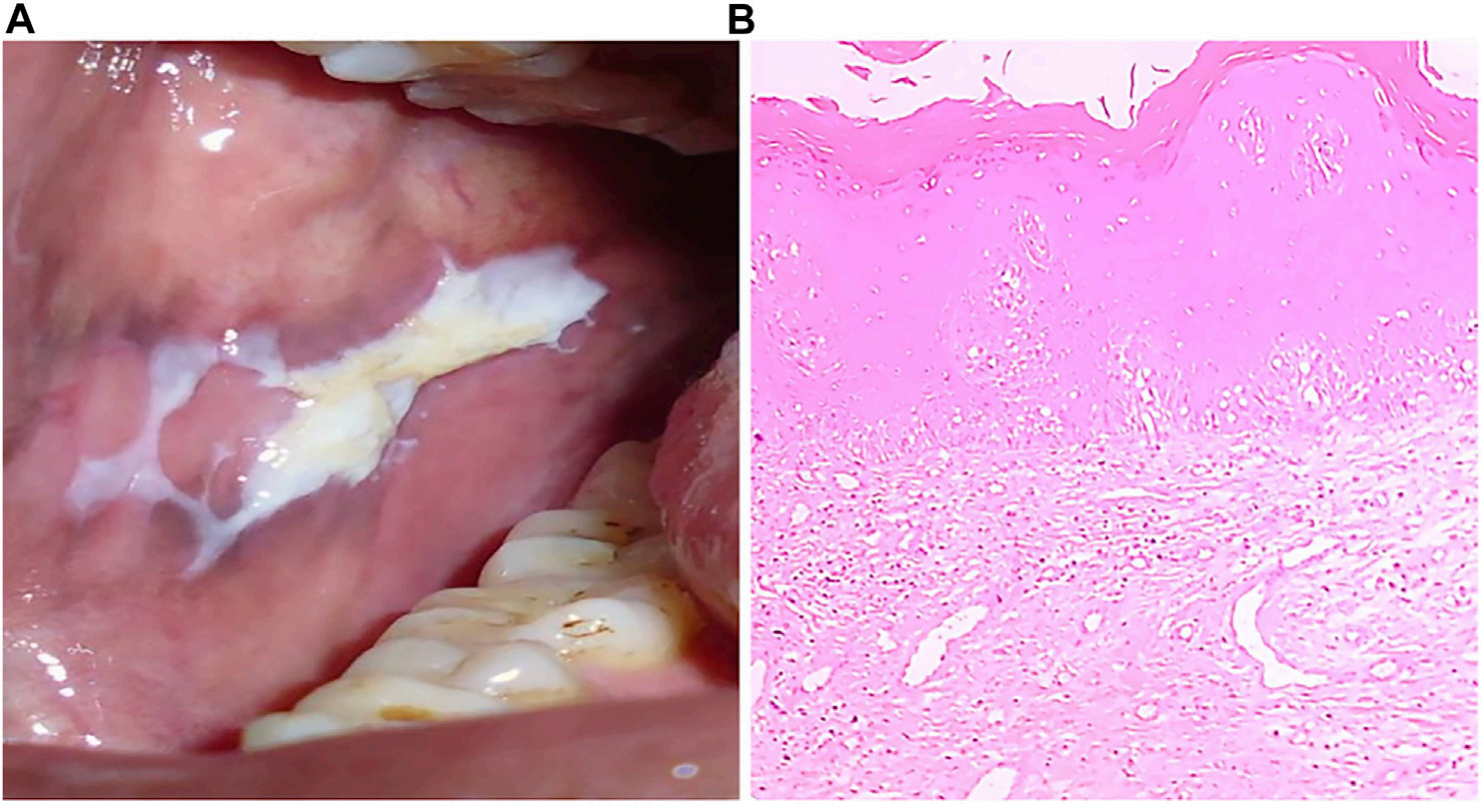
**(A)** Clinical presentation of leukoplakia, a typical appearance of white patch. **(B)** H&E stained section (4x) showing hyperkeratinized stratified squamous epithelium.

The presence of numerous white lesions in the oral cavity mandates the correct identification of leukoplakia. Clinically, leukoplakia can be broadly divided into homogeneous and non-homogeneous sub-groups based on color and morphology (thickness and texture). Homogeneous leukoplakia presents with uniform white patches with thin, flat, or wrinkled surfaces that could possibly show fine cracks or fissures. Other sub-variants of homogeneous leukoplakia were reported to show a velvet or pumice-stone type appearance. Non-homogeneous leukoplakia shows varying degrees of mixed (red-white) appearances, and most common mixed red-white lesions are 1) speckled (also called erythroleukoplakia) and predominantly retain their white character; 2) nodular with small polypoid outgrowths and round red or white excrescences; and 3) verrucous with uniform white patches and wrinkled or ridged surfaces. These lesions are recognized to have a high risk of MT (Van der Waal, 2019; Carrard and Van der Waal, 2018). Proliferative verrucous leukoplakia (PVL) is another sub-type of non-homogeneous leukoplakia that has been characterized by multifocal presentation with surface projections. This is seen in the gingival region but also present in other sites of the oral cavity (Bagan et al., 2003; Gupta et al., 2017). PVL is more common in elderly women and its association with tobacco use is not well explained (Issrani et al., 2013). The clinical characterization of leukoplakia includes aetiological factors (tobacco or areca nut use, idiopathic), anatomical sites, and the size of lesions. Histological characterization is important for definitive diagnosis of leukoplakia if other lesions have been exempted. Leukoplakia presentation can vary from hyperkeratotic lesions to severe dysplasia (Figure 3) (Pindborg et al., 1997). Dysplastic leukoplakia carries higher risk of MT than a non-dysplastic leukoplakia. Epithelial dysplasia can be of mild, moderate, and severe types (Brennan et al., 2007). The MT rate of leukoplakia is reported to be 9.5% (Iocca et al., 2020). The morphological changes during dysplastic changes are listed below (Ranganathan and Kavitha, 2019).

List 1: Histopathological features of epithelial dysplasia

1) Abnormal variation in nuclear size (anisonucleosis) and shape (nuclear pleomorphism)
2) Change in cell size (anisocytosis) and shape (cellular pleomorphism)
3) Loss of polarity of the basal cells
4) Presence of more than one layer of cells having a basaloid appearance
5) Increased nuclear–cytoplasmic ratio
6) Drop-shaped rete processes
7) Irregular epithelial stratification
8) Increased number of mitotic figures (a few abnormal mitoses may be present)
9) Presence of mitotic figures in the superficial half of the epithelium
10) Nuclear hyperchromatism
11) Enlarged nucleoli
12) Reduction of cellular cohesion
13) Keratinization of single cells or cell groups in the prickle layer

These histopathological changes are clinically used as a confirmatory test for leukoplakia positivity and for dysplastic changes (Kramer et al., 1978; van der Waal and Axéll, 2002a). Some exceptional cases have been reported where dysplastic lesions remain clinically unchanged, while MT occurs in non-dysplastic leukoplakia (Arduino et al., 2021). Thus, it is more important to consider the histopathological features instead of keeping them under observation without conducting biopsies. Different classification systems have been put forward for grading epithelial dysplasia. The WHO proposed a five-stage histopathological classification system, such as hyperplasia, mild dysplasia, moderate dysplasia, severe dysplasia, and carcinoma *in-situ*. Other grading systems include the Squamous Intraepithelial Neoplasia (SIN) system, Ljubljana classification of Squamous Intraepithelial Lesions (SIL), and Oral intraepithelial neoplasia (OIN) System. Moreover, dysplasia displays a spectrum of cellular abnormalities such that no existing criteria alone can distinguish different stages. Different structural and cytological parameters are taken into consideration for the diagnosis of epithelial dysplasia (Bouquot et al., 2006; van der Waal, 2009). Histological leukoplakia reports are recommended to include an affirmation on the presence or absence of epithelial dysplasia (or OIN) and an appraisal of its severity. Severe epithelial dysplasia is graded as carcinoma *in situ*.

#### 2.1.5 Management and Treatment

Presently there is no clearly defined clinical management guideline for leukoplakia. The premalignant nature of leukoplakia has emphasized the essentiality of its diagnosis and treatment. The best practice still includes the exclusion of known recognizable lesions and identifying the root cause. Leukoplakia is diagnosed only when the white patches cannot be scraped off or cannot be distinguished clinically or pathologically as other known oral manifestations. These white patches often arise due to local injuries of the oral cavity or due to infection. Various white patches have been identified so far, however, not every identified white patch is leukoplakia. To distinguish leukoplakia and non-leukoplakia white patches, it is most important to define these patches based on clinical, histopathological, and dysplastic features (Villa and Woo, 2017; Carrard and Van der Waal, 2018). A clinical diagnosis of leukoplakia takes into consideration various factors like age, tobacco habit (smoke and smokeless), the onset of disease, duration of disease, lesion size, lesion color, lesion texture, the oral subsite, and medical history (van der Waal and Axéll, 2002b).

However, the recommended diagnostic methods were not so successful in classifying the white patches at the early stage of diagnosis. Therefore, it is recommended now to use the word lesion instead of white patches. Depending on the clinical appraisal and the extent of the white lesions, provisional and definitive diagnostic methods are recommended (Axéll et al., 1996). A provisional (temporary) diagnosis is made when a practitioner fails to recognize the type of oral manifestation and it mainly occurs at the early stage of lesion development. For these types of lesions, certainty factors are assigned to assess the degree of correct identification (van der Waal and Axéll, 2002a; Brouns et al., 2013). A definitive (confirmative) diagnosis is made on the basis of exclusion criteria where identification and elimination of suspected etiological factors associated with other intervening oral manifestations are done (Axéll et al., 1996; van der Waal and Axéll, 2002a). To reach a precise definitive diagnosis for leukoplakia, provisional and histopathological factors are taken into consideration together.

The need for treatment is primarily based on the nature of the leukoplakia lesions. Most of the lesions are asymptomatic. The proposed treatment attempt is to prevent cancer development and to evaluate the clinical/histological resolution of oral leukoplakia (Lodi and Porter, 2008). The proposed interventions include surgical excision with different techniques (scalpel, cryosurgery, photodynamic therapy, laser surgery, and vaporization), medical treatment (topical or systemic), cessation of risk activities (smoking and alcohol), and follow-up (Lodi et al., 2016). Histopathologically identified white lesions with dysplasia (grade 1 and 2) or carcinoma *in-situ* (grade 3) are advised to be excised, especially in moderate or severe epithelial dysplasia cases where the margin is not clear. Non-surgical intervention advocates the use of topical bleomycin, systemic retinoids, and systemic lycopene to treat dysplasia (Ribeiro et al., 2010).

### 2.2 Oral Lichen Planus

#### 2.2.1 Definition

Oral lichen planus (OLP) is recognized as a chronic inflammatory disease of the oral basal epithelium mediated by T-cells. Immune infiltration is a common feature of the OLP, especially CD8^+^ Lymphocytes. The WHO-recommended definition for OLP is “A chronic inflammatory disorder of unknown aetiology with characteristic relapses and remissions, displaying white reticular lesions, accompanied or not by atrophic, erosive and ulcerative and/or plaque type areas. Lesions are frequently bilaterally symmetrical. Desquamative gingivitis may be a feature” (Warnakulasuriya et al., 2020a).

#### 2.2.2 Epidemiology and Etiology

The global prevalence of OLP is estimated to be 1.01%. The geographical prevalence showed that North America and Asia (especially India 0.49%) have the lowest incident rates of 0.47% and 0.83% respectively. Meanwhile, Southern Central America, Africa, and Europe have the highest incident rates of 1.74, 1.43, and 1.32% respectively (González‐Moles et al., 2020). Women are affected more frequently than men (Laniosz et al., 2019). OLP is known as a disease of unknown etiology, however, genetic susceptibility, immunological illnesses, malnutrition, psychological, and infection are considered probable causative factors (Torrente Castells et al., 2010).

#### 2.2.3 Pathogenesis

**FIGURE 4:**
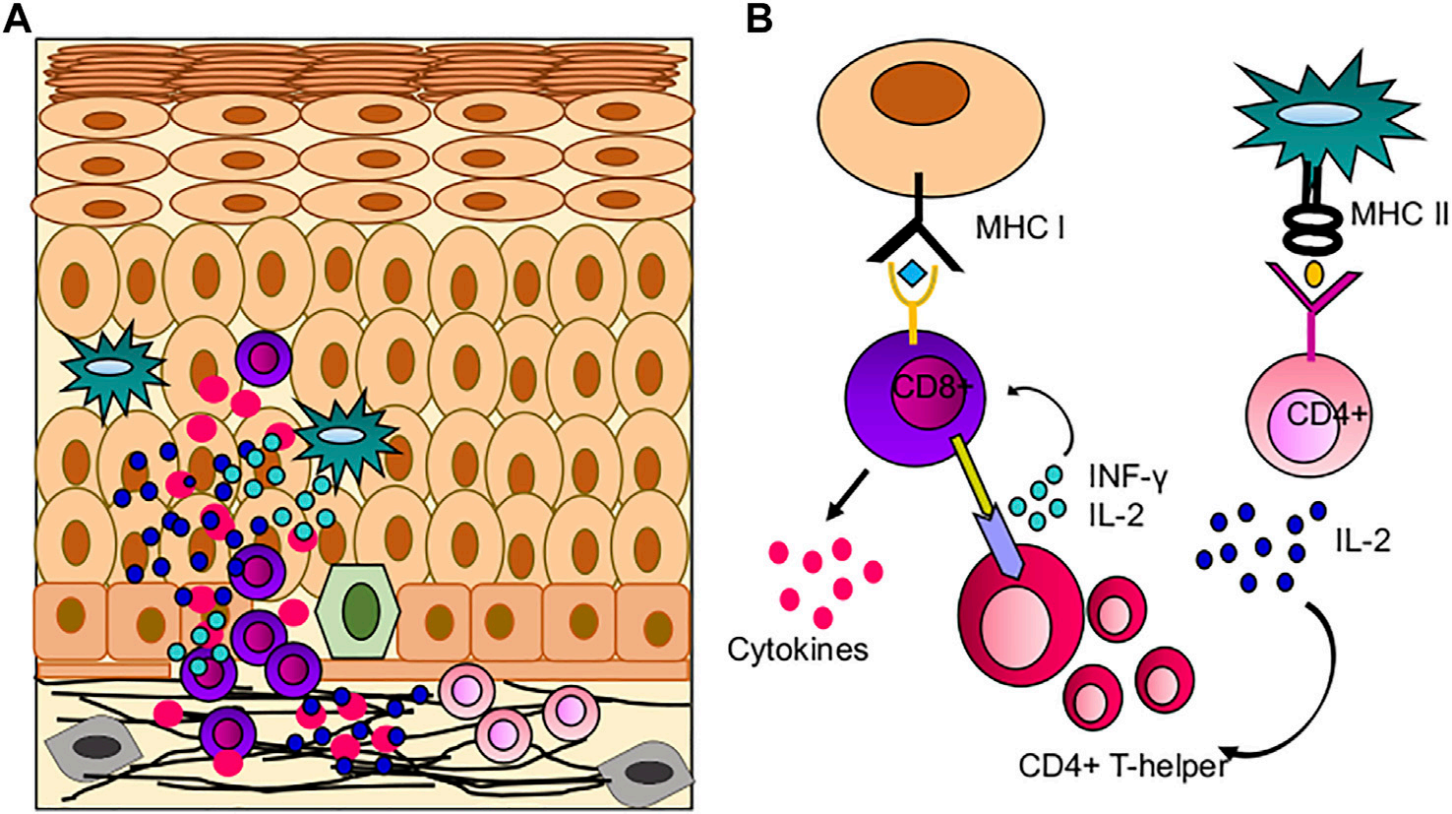
Role of immune cells in OLP pathogenesis; **(A)** Immune infiltration in OLP, CD8^+^ T cells mediated hyperimmune activation leading to apoptosis of basal keratinocytes. **(B)** Possible mechanism of hyperimmune response in buccal mucosa, where CD8^+^ T cells are activated by oral keratinocytes and CD4^+^ T cells.

Since OLP is associated with immune infiltration, the immunopathogenesis of OLP has been studied mainly in erosive and reticular forms (Kurago, 2016). Expression of Th1 and Th2 cytokines is observed in OLP lesions and tissue secretions (Sugerman and Savage, 2002). Infiltrating cytotoxic CD8^+^ T cells promote the apoptosis of the basal cells of the oral mucosa resulting in autoimmunity. CD8^+^ T cells are activated by the major histocompatibility complex (MHC)-1 on keratinocytes or indirectly by antigen-presenting cells through CD4^+^ lymphocytes (Th1 subset) (Figure 4) (Lavanya et al., 2011). CD4^+^ T helper subsets are present in the sub epithelium and lamina propria in OLP lesions, and these CD4^+^ T cells can differentiate into different subsets, including Th1, Th2, Th9, and Th17 cells, depending on the cytokine environment, and may thus further amplify the immune response (Rengarajan et al., 2000; Vignali et al., 2008). Increased Th9 levels in reticular- and Th17 levels in erosive-type OLP are also reported. In this milieu, cytokines such as IL-4 and IL-6 may play critical roles in the CD4^+^ T cell differentiation into Th9 and Th17 respectively in local OLP lesions (Wang et al., 2017). TLR-2 immunoexpression in the OLP epithelium is significantly higher in comparison to the normal buccal mucosa, suggesting that TLR-2 may be involved in the OLP pathogenesis (Amin et al., 2020). A CD34 a marker of angiogenesis is found to be more strongly overexpressed in OLP than in normal buccal mucosa, especially in the erosive form compared to the reticular form, indicating increased angiogenesis (Amin et al., 2020). Basal and parabasal keratinocytes showed positive p53 expression that significantly correlates with the degree of dysplasia in OLP (Mithani et al., 2007; Hadzi-Mihailovic et al., 2017). Loss of c-ErbB2 activity in OLP is an important indicator of neoplastic transformation and carcinogenesis (Parise Junior et al., 2004). Other biomarkers showing promising results in pathogenesis were also recognized, such as apoptosis regulators (p53, MCL-1), cell cycle regulators (BMI1, p16), extracellular matrix regulators (MMP-2, 3 and 9), and inflammatory molecules (TNF-α, IL-6, and COX-2) (Caruntu et al., 2018). EMT‐related proteins claudin‐1, claudin‐4, claudin‐7, E‐cadherin, TWIST1, and ZEB1 were also reported to be deregulated in OLP. Claudin‐1, claudin‐4, and E‐cadherin levels were significantly reduced in OLP in comparison to the healthy oral mucosa. The expression of claudin protein shows epithelial stratification, for example, claudin-1 is absent in the basal layer and stroma in OLP, claudin-7 is present more in stroma of OPL. E-cadherin is more localized to the intermediate level of oral epithelium in OLP than basal and stroma, suggesting the loss of intercellular connections and basal keratinocytes may facilitate T-cell migration to epithelium (Hämäläinen et al., 2019). In oral cancer, loss of E-cadherin greatly enhances the invasion and metastatic potential. DNA mutation, transcriptional control, and promoter methylation are common mechanism causing loss of E-cadherin expression (Pannone et al., 2014). Genetic variation study reported chromosomal instability in 3p, 9p and 17p in OLP. Monosomy of chromosome 9 is more prevalent and trigger the MT of OLP (Mithani et al., 2007).

#### 2.2.4 Clinical and Histological Presentation

**FIGURE 5:**
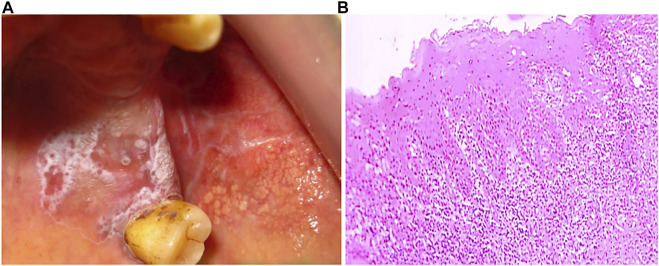
**(A)** Oral lichen planus **(B)** Hyperkeratinized stratified squamous epithelium (10x) showing basal cell degeneration at focal areas. Juxtaepithelially stroma shows presence of lymphocytes infiltration.

Broadly, lichen planus can be classified into cutaneous lichen planus (CLP) and mucosal LP (mainly involving oral mucosa) (Gorouhi et al., 2014). Clinically OLP is of six types *viz.* reticular, atrophic, plaque-like, papular, erosive/ulcerative, and bullous (Edwards and Kelsch, 2002; Mollaoglu, 2000). Reticular and erosive OLP is the most common and widely studied (Oliveira Alves et al., 2010). Reticular OLP is also known as Wickham’s striae due to its white lacy streak-like appearance encircled by distinctive erythematous (red) borders. The white stretches are located bilaterally on the posterior jugal mucosa, tongue, gums, and palate (Figure 5A). Erosive OLP is presented as erythematous and atrophic areas surrounded by thin radiating keratotic striae. In desquamative gingivitis, atrophy and ulceration are mainly restricted to the gingival mucosa and are accompanied by regular pain to severe discomfort that can interfere with chewing. Plaque-like OLP and leukoplakia shows similarities in clinical presentation, showing as large white homogenous patches. Papular OLP is rarely observed and is characterized by small white papules of pinpoint size and are asymptomatic. The ulcerative subtype shows atrophic lesions with erythema background and superficial white striae at the margins. Thus, it is a combination of two clinical presentations named atrophic-erosive lichen planus. It primarily affects the gingiva and buccal mucosa region, particularly the areas surrounding the second and third molar teeth. Bullous is the most unusual clinical form and mostly involves salivary gland ducts, where lymphocyte infiltration causes blisters formation and increases the size, which, upon rupture, causes ulceration and is painful. Nikolsky’s sign may be positive (Sousa and Rosa, 2008; Gorouhi et al., 2014; Gupta and Jawanda, 2015).

The histopathologic features of OLP include hyperkeratosis of the epithelium, liquefaction degeneration of basal layer accompanied by apoptosis of the keratinocytes, formation of a dense band-like structure due to lymphocytic infiltrate in the superficial lamina propria, atrophy of spinous layer, saw-tooth epithelial ridges, a homogeneous eosinophilic deposit at the epithelium-connective tissue junction (Figure 5B) (Van Der Meij and Van Der Waal, 2003; Cheng et al., 2016). Degenerating keratinocytes appear as colloid bodies (Civatte bodies) in the lower epithelial layer. Increased immune reaction facilitates the degeneration of the basal layer, disrupts the connection between the basement membrane and basal layer (e.g., hemi desmosomes, filaments, and fibrils), and incapacitates the epithelial connective tissue interface. The loss of cellular connectivity may result in histologic cleft (Max-Joseph spaces) formation and blisters on the oral mucosa (bullous LP), which can be easily identified during a clinical examination. B cells and plasma cells may also be found, though they are not very common (Gupta and Jawanda, 2015; Chiang et al., 2018).

#### 2.2.5 Management and Treatment

OLP represents a variety of surface morphology such as white striations, white papules, white plaques, erythema, erosions, or blisters primarily present in the buccal mucosa, tongue, and gingivae. In OLP, lesions have bilateral and symmetric distribution. Diagnosis of OLP has become challenging due to the presence of overlapping features and inflammatory infiltrate with those of other oral lesions (OLLs) (Speight et al., 2018). In most cases, histopathologic evaluation of lesion tissue is required to obtain a definitive diagnosis except for reticular OLP or where cutaneous lesions are present. Therefore, a biopsy is done to establish baseline histopathologic features (Edwards and Kelsch, 2002). The differential diagnosis involves exclusion of common OLP-like lesions, which includes cheek chewing/frictional keratosis, lichenoid reactions, leukoplakia, lupus erythematosus, pemphigus, mucous membrane pemphigoid, erythematous candidiasis, chronic ulcerative stomatitis, and graft vs. host disease (Lavanya et al., 2011). Among differential diagnoses of OLP, drug lichenoid reactions (DLR) are more difficult to distinguish virtually from OLP both clinically and histopathologically. Many drugs have been listed to be involved in DLR: anticholinergics, anticonvulsants, antidiabetics, antidiarrheals, antimycobacterials, antihistamines, antifungals, antimalarials, antiretrovirals, and antigout, anti-inflammatory, antibiotic, antihelminthic, and antiparkinsonian drugs, chemotherapy drugs, cardiovascular drugs, diuretics, immunomodulatory and biological therapies, lipid lowering drugs, psychiatricdrugs, retinoids, and other medications (Gorouhi et al., 2014). The widely accepted method to diagnose lichenoid drug reactions is to study the effect of the drug reaction after withdrawal and readmission in the patient to observe for any sign of discomfort. Dental restorative material, such as silver-mercury amalgam fillings mainly restricted to old and corroded dental fillings, can also induce DLR (Athavale et al., 2003; Lartitegui-Sebastián et al., 2012).

Management includes the stipulation of the diagnosis method, depending on patient’s history, clinical, and, histopathology report, direct immunofluorescence (DIF), indirect immunofluorescence (IIF), and cutaneous patch testing. The initial cure is focused on the alleviation of painful symptoms, use of medication if necessary, and healing of ulcerative lesions to decrease the risk of oral cancer transformation as well as a symptom-free period and the maintenance of good oral hygiene and dental status (Potts et al., 1987; Rotaru et al., 2020). MT is mostly seen in atrophic or erosive OLP. Overall MT for OLP is 1.4% (Iocca et al., 2020). Currently, there is no precise cure for OLP, however, systemic treatment is indicated based on symptoms and clinical presentation like, corticosteroids are given to manage the lesions. In case of asymptomatic, non-ulcerative lesions follow-up is suggested. Atrophic/erosive or ulcerative lesions are painful and have a poor prognosis, while the reticular form has the best prognosis. Before initiating treatment, it is important to rule out candidiasis, as it may aggravate an existing candida infection. Medication is suggested in case of severe pain or it is recommended to maintain oral hygiene during periods of active disease. For oral hygiene, the use of antibacterial mouthwash, e.g., chlorhexidine, is recommended (Rotaru et al., 2020).

OLP therapies include drug therapy, surgery, psoralen with ultraviolet light A (PUVA), and laser treatment. Topical and systemic drugs have been prescribed for OLP treatment. Topical agents such as analgesic (lignocaine gel or dyphenhydramine), steroids (triamcinolone, betamethasone or fluocinolone acetonide, Clobetasol proprionate), cyclosporin A, and retinoic acid are used. Systemic medications include corticosteroids (prednisolone), tretinoin, and isotretinoin. Systemic corticosteroids are preferably used in erosive OLP or multi-site disease. Moreover, topical corticosteroids are extensively used in the treatment of OLP to subdue pain and inflammation. The effectiveness of topical corticosteroids is higher than systemic corticosteroids. In cases where OLP unresponsive to topical steroids or patients with mucocutaneous disease, it is often advised to switch to systemic corticosteroid use (Snyder et al., 1982; Silverman et al., 1985). Fluorinated steroids are advised in case of failure of other medication. It is used as a formulation of 0.05% fluocinonide and 0.1% fluocinolone acetonide. Fluocinolone acetonide is shown to be more potent than 0.1% triamcinolone acetonide preparation with fewer side effects (Silverman et al., 1985). The use of zinc acetate 50 mg twice daily was found to be effective in decreasing the burning sensation, pain, and lesion size in symptomatic OLP (Silverman et al., 1985). Topical 0.1% vitamin A have rapid action on OLP, however, relapse has been reported after 2–5 weeks of treatment discontinuation (Ebner et al., 1973).

Dysplastic lesions are excised using surgery, cryotherapy, CO_2_ laser, and ND:YAG laser. Photochemotherapy with 8-methoxypsoralen and long-wave ultraviolet light (PUVA) has been adopted for recalcitrant erosive OLP treatment. However, some side effects were reported, including nausea, dizziness, eye symptoms, paraesthesia, and headache. Moreover, PUVA with topical 0.01% trioxsalen has been shown to have no side effect (Boorghani et al., 2010).

### 2.3 Oral Submucous Fibrosis

#### 2.3.1 Definition

Oral submucous fibrosis (OSMF) is a fibrotic condition of oral mucosa characterized by epithelial immune cell infiltration followed by a fibro-elastic change in the lamina propria and submucosa leading to stiffness of the oral mucosa (Abidullah et al., 2018). The clinical definition describes it as “a debilitating, progressive, irreversible collagen metabolic disorder induced by chronic chewing of areca nut and its commercial preparations; affecting the oral mucosa and occasionally the pharynx and oesophagus; leading to mucosal stiffness and functional morbidity; and has a potential risk of malignant transformation” (More and Rao, 2019).

#### 2.3.2 Epidemiology and Etiology

The overall prevalence of OSMF is 4.96%. OSMF shows geographical stratification and is confined to South and South East Asia. It is mainly associated with the habits of chewing betel quid/areca nuts. It is reported that about 10%–20% of the world’s population are of these habits, especially in countries like India and Taiwan. More than 90% of OSMF patients were found to be betel-quid chewers (Kujan et al., 2020). In India, areca nuts are chewed directly or are available in various commercial forms like supari, mawa, paan masala, and betel quid with or without tobacco (Sinor et al., 1990).

#### 2.3.3 Pathogenesis

**FIGURE 6:**
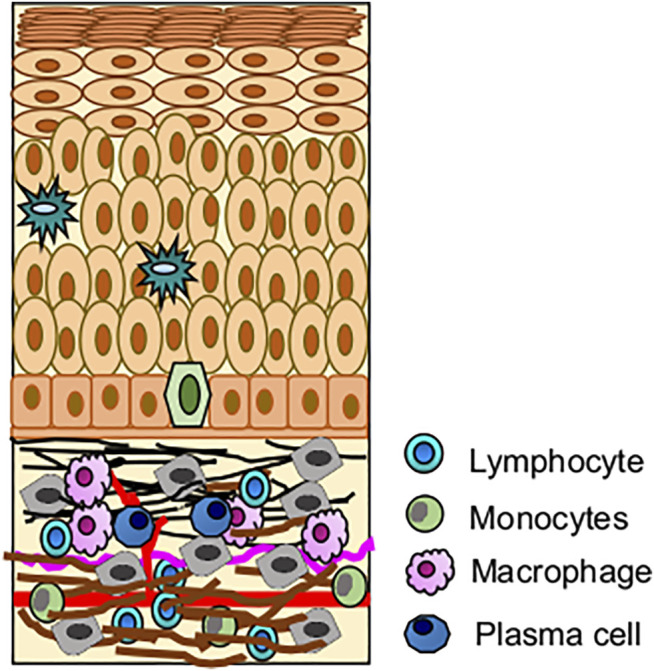
Hyperkeratotic epithelial cells in OSMF. Immune activation in OSMF show the presence of different types of immune infiltrates in submucous layer. Increase in fibroblast cells leads to high production of collagen fibres (brown bar) leading to fibrosis of oral mucosa.

**FIGURE 7:**
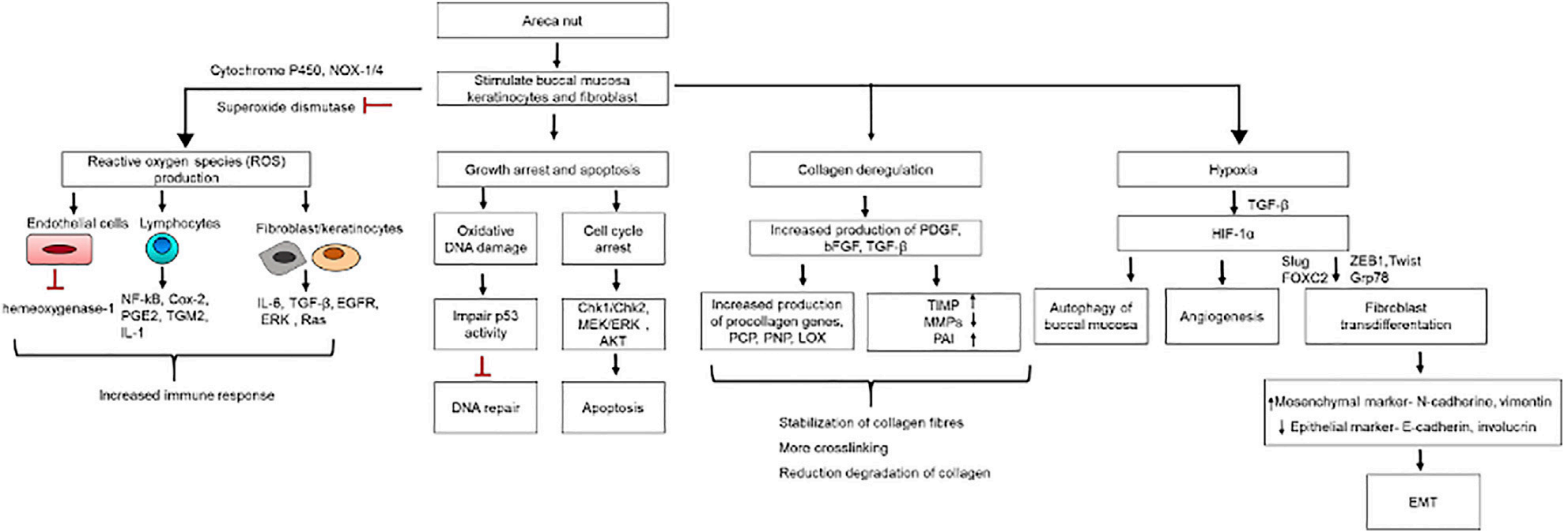
OSMF pathogenesis: Cellular and molecular changes in buccal mucosa due to areca nut exposure.

It is believed that a number of factors are associated with the pathogenesis of OSMF. The possible identified factors include areca nut products, chilies, genetic and immune reactions, and nutritional deficiencies. Among identified factors, areca nuts have been recognized as one of the most important risk factors. Moreover, numerous studies elucidating etiology and pathogenesis seem too focused on extracellular matrix (ECM) modulation. OSMF symptoms include ulceration, xerostomia, a burning sensation, and restricted mouth opening due to collagen dysregulation, i.e., increased biosynthesis and reduced clearance (Sinor et al., 1990; Angadi and Rekha, 2011). Disrupted collagen metabolism was identified as the most fundamental cause of OSMF (Figure 6). Arecoline, a major constituent of the areca nut, acts as an initiator of OSMF pathogenesis, primarily causing increased production of collagen proteins (Illeperuma et al., 2015a). Other metabolites present in areca nuts, such as arecadine, guvacoline, and guvacine, also contribute to OSMF. Arecoline under the influence of slaked lime [Ca (OH)_2_] is hydrolyzed to arecadine, which has pronounced effects on collagen (Tilakaratne et al., 2006; Tsou et al., 2019). Flavonoid components (tannins and catechin) of areca nuts inhibit collagenase activity and thus have a direct influence on collagen metabolism. These metabolites are a constant source of irritation of oral tissues, and areca nut fibers cause mechanical injury through friction from chewing. In combination, this can result in the initiation of juxta-epithelial inflammatory cell infiltration (Hoffmann et al., 1994; Chiang et al., 2002). Inflammation is well characterized by the presence of T cells, macrophages, prostaglandins (Pgs), IL-6, tumor necrosis factor (TNF), INF-α, and TGF-β. TGF-β is a key regulator of ECM assembly and remodeling. TGF-β increases the production of collagen fibers by increasing the transcription of procollagen genes (COL1A2, COL3A1, COL6A1, COL6A3, and COL7A1), which are further cleaved by procollagen C-proteinase (PCP) and procollagen N-proteinase (PNP) to produce soluble collagen fibrils. These fibrils are stabilized by LOX to covalently produce cross-linked mature fibril forms, which are resistant to proteolysis. LOX activity is dependent on copper and areca nuts are source of copper, thus increasing the soluble collagen level. TGF-β also reduces the collagen degradation by inhibiting matrix metalloproteinases (MMPs) activity by activating tissue inhibitor of matrix metalloproteinase gene (TIMPs), thus stabilizing collagen in ECM. Plasminogen activator inhibitor (PAI) inhibits tissue plasminogen activator (tPA) and urokinase plasminogen activator (uPA) and is involved in the conversion of plasminogen (Plg) into active plasmin. It also induces fibrosis in OSMF by blocking the plasmin mediated MMPs activation. Thus, overall TGF-β is involved in the stabilization of collagen fibers in the ECM, increasing the collagen disposition in oral tissue and leading to fibrosis (Tilakaratne et al., 2006; Rajalalitha and Vali, 2005; Khan et al., 2012; Angadi and Rao, 2011). About 7–13% OSMF cases are reported to progress into oral cancer (Illeperuma et al., 2015b). Areca nut exposure of the oral epithelium may trigger oxidative DNA damage and DNA, DSB, and cytokine‐mediated ROS generation, which might contribute to the MT of OSMF (Figure 7) (Illeperuma et al., 2015a). At the molecular level, irregularities in the function of O6-methylguanine-DNA methyltransferase, p53, p16^INK4alpha^/p19^ARF^, C-JUN, HSP70, HSP27, and mitochondrial DNA mutation has been identified to promote the MT (Wollina et al., 2015a).

Genomic instability is present in 47%–53% of the OSMF samples. A total of 23 hot-spot LOH loci have been identified in 14 chromosomes (chromosome 1, 3, 4, 6, 7, 9, 10, 11, 12, 13, 15, 18, 19, and 20). Among 23 hot-spots, chromosome 13 contains the largest LOH regions starting from 13q14 to 13q33 (according to chromosomal size). The 13q region is highly susceptible to genomic instability in HNSCC, and this region contains genes such as AKAP11, PCDH9, GPC6, UCHL3, and LMO7. Other genomics regions containing genes involved in various functions like hypoxia, inflammation, toxin metabolism, DNA repair, ubiquitin pathway, cell adhesion, and migration, and these are also implicated in OSMF (Teh et al., 2008).

#### 2.3.4 Clinical and Histological Presentation

**FIGURE 8:**
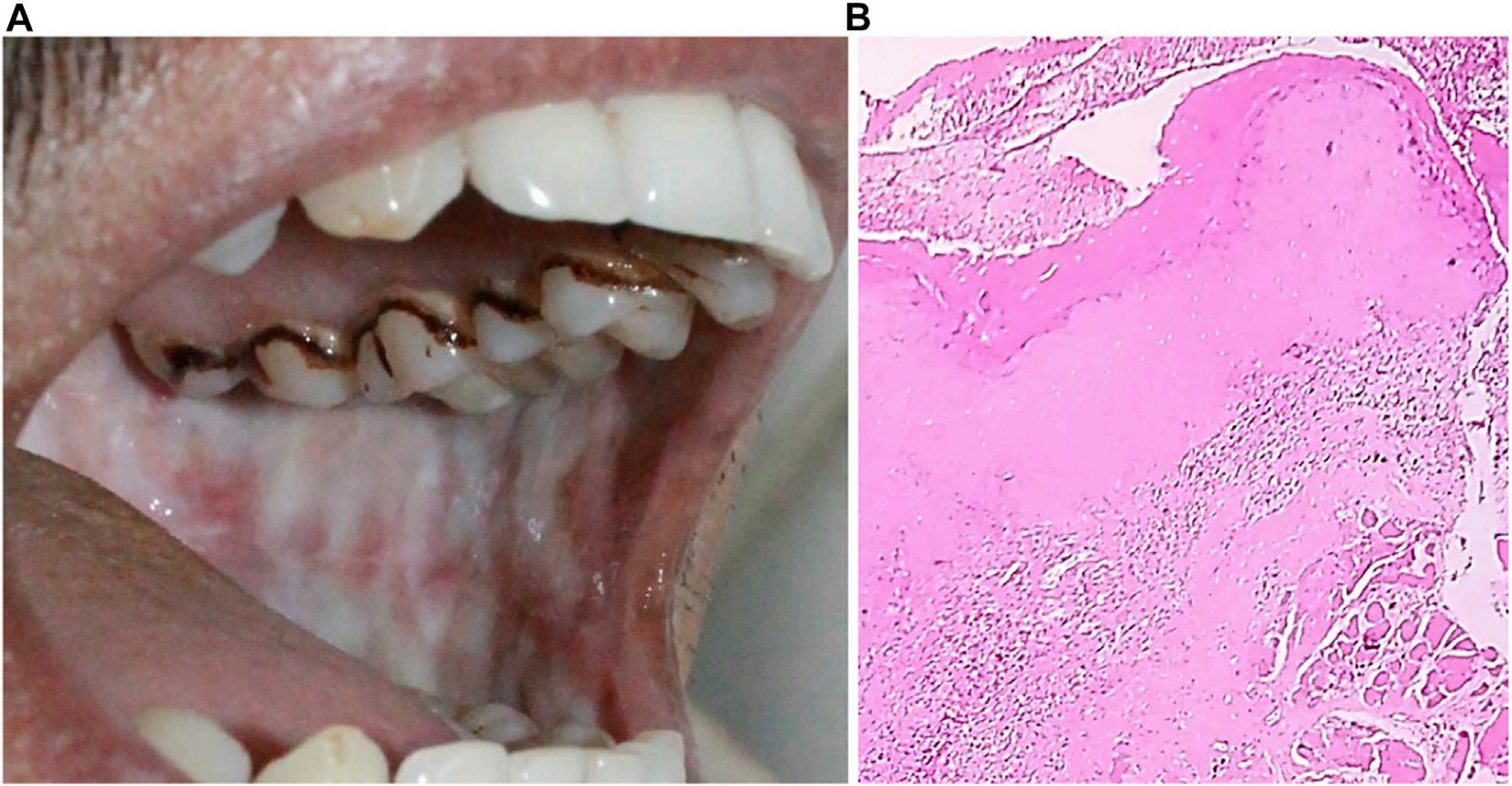
**(A)** Oral submucosal fibrosis of cheek mucosa. **(B)** Keratinized stratified squamous epithelium (4x) showing presence of hyalinization and collagenous connective tissue stroma.

Clinical diagnosis involves the identification of oral lesions, restricted tongue movement and depapillation, blanching and leathery texture of oral mucosa, loss of pigmentation of the oral mucosa, and gradual reduction in mouth opening (Figure 8A) (More et al., 2012; Priyadharshni, 2014). Clinical classification helps in the management and treatment of OSMF patients. Clinically, OSMF can be classified into three stages: early, moderate, and advanced (Rao et al., 2020). Various classification systems have been put forward for OSMF diagnosis and management. Recently, Arakeri G. et al. proposed a three-component classification system TFM (trismus, fibrosis, and malignant transformation), which allows for a distinction between medical, surgical, and malignant disease therapy (Arakeri et al., 2018). The main histopathological attribute of OSMF is collagen deposition within the submucosa and the epithelial morphology modification-like appearance of the rete-peg shapes (Figure 8B). The presence of epithelial alterations in the different stages of OSMF likely range from atrophy with hypoplasia to hyperplasia and/or dysplasia. Histopathological grading involves immune infiltration count, epithelial thickness, collagen proportionate area, and blood vessel parameters. With an advanced stage, there is a decrease in immune localization and inflammation, whereas collagen density and epithelial thickness increases (Ramadoss et al., 2021).

#### 2.3.5 Management and Treatment

Clinical diagnosis involves the presence of one or more symptoms followed by a histopathological evaluation to identify any sign of epithelial atrophy with loss of rete ridges and hyalinization of the lamina propria and the underlying muscle (Wollina et al., 2015b).

No available treatment for OSMF is effective, although some conservative surgical interventions may result in improvement. Oral mucosa burning sensations and progressive trismus obstruct normal oral function and further add to the list of complications in the treatment of OSMF patients. The treatment aims at alleviating the symptoms and the cessation of fibrosis progression. Currently, three treatment modalities have been practiced: medical therapy, surgical therapy, and physiotherapy. Moreover, the treatment of OSMF depends on the degree of disease progression. At the initial stage, stopping habits and nutritional supplements are recommended, at moderate stages, conservative treatment such as intra-lesional injections along with medical treatment is provided, and at advanced stages, surgical interventions are needed. The non-surgical methods include the use of medication, vitamin and iron supplements, and a diet rich in minerals (Passi et al., 2017; Shih et al., 2019).

Hyperbaric oxygen therapy (HBOT), where hyperbaric chambers with ambient oxygen pressure usually higher than atmospheric pressure are used to treat the patients. HBOT reduces inflammation by decreasing the production of pro-inflammatory cytokines such as IL-1, IL-6, and IL-10 (Weisz et al., 1997). It also enhances fibroblast apoptosis by reducing IL-1β and TNF-α levels (Oscarsson et al., 2017). Reactive oxygen species such as E-SOD, GPx, catalase, paraoxonase, and heme-oxygenase-1 are also inhibited by HBOT (Ozden et al., 2004). Thus, HBOT exhibits therapeutics effect on OSMF by suppressing fibroblast activity, increasing anti-inflammatory and antioxidant properties (Shih et al., 2019).

Corticosteroids are immunosuppressive agents used as anti-inflammatories to alleviate the pain and burning sensation (Borle and Borle, 1991). Proteolytic enzymes like hyaluronidase, collagenase, and chymotrypsin are used to dissolve extracellular matrix components such as hyaluronan and collagen. They are usually given in combination with triamcinolone or dexamethasone (Daga et al., 2017). Pentoxifylline is also prescribed as an adjunct therapy in the routine management of OSMF. Together, these prevent the immune response by increasing microcirculation and reducing platelets aggregation, granulocyte adhesion, and degranulation of neutrophils, enhancing natural killer cell activity, and inhibiting T-cell and B-cell activation. It is helpful in reducing the fibrosis of the skin and improving mouth opening and tongue protrusion. (Rajendran et al., 2006; Patil and Maheshwari, 2014; Sadaksharam and Mahalingam, 2017). Placental extracts are also used as immunosuppressive because of their antioxidant and anti-inflammatory activity (Kisave et al., 2020). Supplements rich in vitamins, minerals, and antioxidants are recommended. Lycopene, a powerful antioxidant present in tomatoes, also shows significant improvement. Deficiencies in iron and vitamin B are suggested to play important roles in the pathogenesis of OSMF (Tilakaratne et al., 2006). Interferon-γ has an anti-fibrotic effect, and intralesional injection of recombinant human INF-γ showed improvement in mouth opening and reducing burning sensation (Haque et al., 2001). Traditional Chinese medicines like glabridin, asiatic acid, tanshinone, and salvianolic acid B have shown potential efficacy against OSMF. They downregulate α-SMA, fibronectin, and type I collagen A1 and reduce myofibroblast bioactivity. Studies have shown that they have antioxidant and anti-inflammatory properties and also inhibited collagen biosynthesis and increased collagen degradation (Shih et al., 2019).

In advanced trismus conditions, surgical intervention in OSMF is recommended. It involves the excision of fibrotic bands either with a scalpel or through laser treatment. The surgical approach involves 1) excision of fibrotic bands with scalpel or lasers; 2) reconstruction of the mucosal defect using flaps, grafts, and collagen membranes; 3) adjunctive procedures intraoperatively, including coronoidectomy and masticatory muscle myotomies; and 4) post-operative oral physiotherapy, dietary supplementation and other medications (Kamath, 2015). Physiotherapy includes stretching of oral muscles by using gags either in the conscious or anesthetized patient, significantly improving mouth opening in OSMF patients (Srikrishna and Kumar, 2019).

### 2.4 Erythroplakia

#### 2.4.1 Definition

Erythroplakia is present as a red patch with granular plaque. The WHO definition of oral erythroplakia is “any lesion of the oral mucosa that presents as bright red velvety plaques which cannot be characterized clinically or pathologically as any other recognizable condition” (Kramer et al., 1978).

#### 2.4.2 Epidemiology and Etiology

The prevalence of erythroplakia is rare, with an overall estimate of 0.17% (Mello et al., 2018). It most frequently affected age groups between 40 and 70 years of age. Most affected areas include the tongue (59.1%) and buccal mucosa (22.7%) (Shi et al., 2019). Predisposing factors are unknown, but heavy alcohol consumption and tobacco use are recorded in most of the cases (Hashibe et al., 2000).

#### 2.4.3 Pathogenesis

A high prevalence of p53 mutation is reported in erythroplakia with a frequency of 46% (Qin et al., 1999). Genetic alteration, like Polysomy of chromosomes 7 and 17 (Hittelman et al., 1993), LOH, or allelic gain at 9p, 3p, within the Rb, p53, or DCC gene region have been implicated in erythroplakia (Partridge et al., 1998; Mithani et al., 2007).

#### 2.4.4 Clinical and Histological Presentation

**FIGURE 9:**
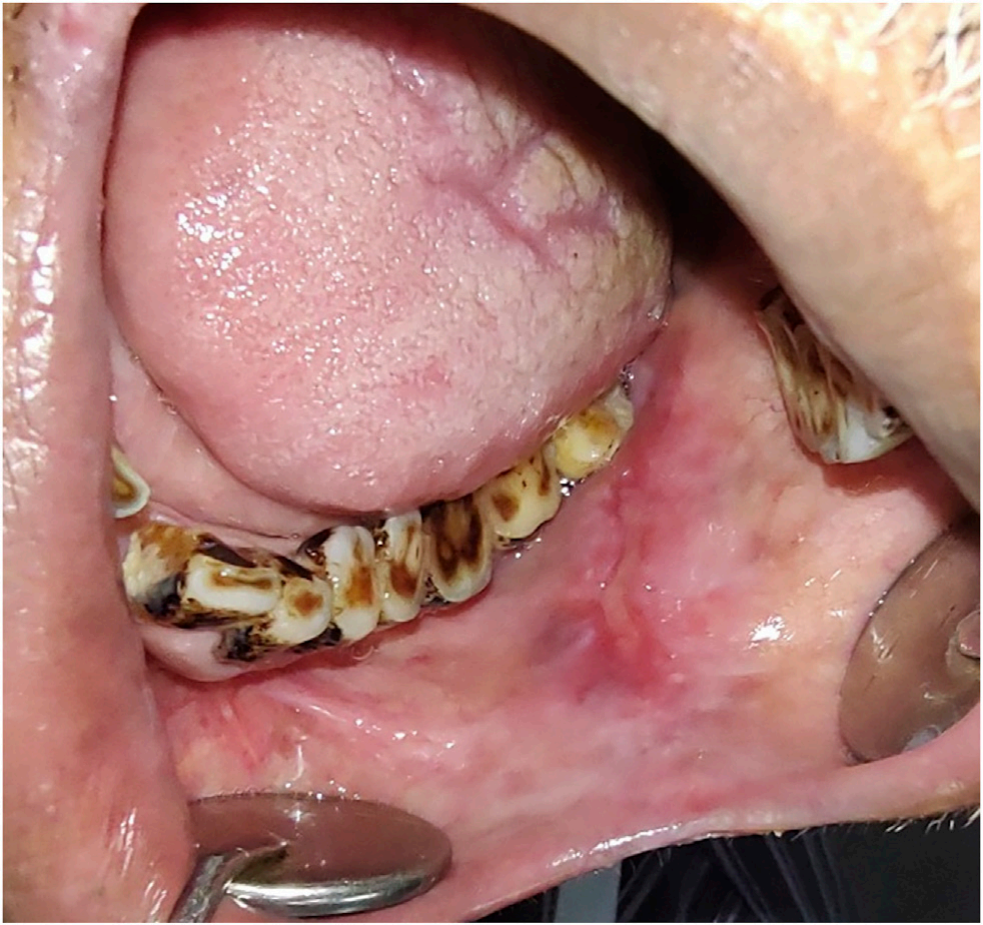
Erythroplakia patch.

Erythroplakia sometimes present as smooth, granular, or nodular patches with a clear margin separation from the surrounding normal oral mucosa. Even though the erythroplakia have smooth and velvety surfaces, other morphological characteristics can also be seen (Villa et al., 2011). These can include the presence of red granular patches with irregular surfaces interspersed with white or yellow spots, referred to as granular erythroplakia. Multiple small irregular white patches scattered in the erythroplakia are also identified, and these lesions are called speckled leukoplakia. Mixed red and white patches of speckled leukoplakia are also known as erythroleukoplakia (Figure 9) (Shafer and Waldron, 1975; Warnakulasuriya, 2000). These are predominantly spotted in the floor of the mouth, soft palate, ventral tongue, and tonsils without any other unusual symptoms. However, some patients may complain about burning sensations (Warnakulasuriya, 2000; Holmstrup, 2018). Interestingly, a lot of studies have been done on the malignant transformation of leukoplakia while erythroplakia, which has higher malignant potential than the white lesions, is less studied. MT of erythroplakia is reported to be 33.1% (Iocca et al., 2020). Erythroplakia is often diagnosed at the late dysplastic stage, and at the time of biopsy it is present as “carcinoma *in situ”* or “invasive carcinoma” (Reichart and Philipsen, 2005). Histological diagnoses assess the severity of epithelial dysplasia. Shafer adopted three levels of dysplasia assessment: mild to moderate, severe to carcinoma *in situ*, and carcinoma. Here, 51% erythroplakia cases harbour carcinoma, severe dysplasia or CIS (Carcinoma *in situ*) are present in 40% of cases, and mild to moderate dysplasia is present in 9% of the cases (Shafer and Waldron, 1975). A study on the Chinese population reported 25% of mild/moderate dysplasia cases, 47.7% of severe dysplasia cases, and 27.3% cases of carcinoma during erythroplakia diagnosis (Shi et al., 2019). A thin epithelial keratinization layer, epithelial atrophy, and dysplasia confirm the presence of erythroplakia (Patait et al., 2016).

#### 2.4.5 Management and Treatment

Erythroplakia diagnosis is based on exclusion criteria where the clinician must rule out all other erythematous conditions for differential diagnosis of red lesions (Reichart and Philipsen, 2005). Other oral lesions considered during the differential diagnosis include erythematous candidiasis, early SCC, local irritation, mucositis, lichen planus, lupus erythematosus, drug reaction, and median rhomboid glossitis. Epithelial atrophic and lack of keratinization are also present. Sometimes hyperplasia is seen (Villa et al., 2011). The definitive diagnosis involves toluidine blue staining prior to incisional biopsy. Excisional biopsy is recommended in severe dysplastic or carcinoma *in situ* cases, while timely follow-up is suggested for histologically moderate or no dysplastic lesions. CO2 laser excision methods are safe and effective due to less postoperative complications like no wound bleeding, trismus, or difficulty in chewing, swallowing, or articulation (Yang et al., 2015).

### 2.5 Proliferative Verrucous Leukoplakia

#### 2.5.1 Definition

PVL is an aggressive form of leukoplakia. It was first described by Hansen et al., in 1985. Initially present as a non-dysplastic keratosis, after a long run (sometimes 20 or more years) it eventually develop into a confluent, multifocal oral keratosis (Murrah and Batsakis, 1994). The WHO defines PVL as a “Progressive, persistent, and irreversible disorder characterized by the presence of multiple leukoplakias that frequently become warty” (Warnakulasuriya et al., 2020a).

#### 2.5.2 Epidemiology and Etiology

PVL is more common in women than men (4:1) (Abadie et al., 2015). The most frequently affected site includes the lower gingiva, buccal mucosa, and tongue (Bagan et al., 2003). The etiology of PVL remains unclear, however, tobacco is ruled out as an etiological factor as PVL has been seen in both smokers and non-smokers (Abadie et al., 2015). HPV presence was also detected in PVL, 89% of PVL samples were HPV positive, mainly HPV 16 (Palefsky et al., 1995). Similarly, 60% of PVL samples were detected EBV positive (Began et al., 2008). The Highest MT rate has been seen in PVL 49.5% in comparison to other OPMDs (Iocca et al., 2020).

#### 2.5.3 Pathogenesis

Genetic alteration involves a change in ploidy level (Klanrit et al., 2007). Aneuploidy is present in 89.2% of the PVL (Gouvêa et al., 2013). LOH is the most frequent alteration in PVL. Allelic loss at 9p21 was present in 63.2% of cases. INFα, D9S1748, and D9S171 are located in 9p21 loci. Loss of one or more of these markers was reported in PVL patients: approximately 47% of cases were reported to have a loss of a single marker, 10.5% have a loss of two markers, and 5.3% cases have a loss of all three markers. Allelic loss of D9S171 (45.5%) is most frequent followed by INFα (35.3%) and D9S1748 (26.3%). In total, 40% of PVL cases show homozygous deletion of p14^*ARF*^. The subsequent loss of p14^*ARF*^ and D9S1748 is observed in 60% of PVL cases. In total, 45% of cases were associated with the loss of both p16^*INK4a*^ and p14^*ARF*^, and 85% of the PVL lesions were detected to be p53 positive, though no association between p53 and INK4a/ARF in the PVL tissues were detected (Kresty et al., 2008). Higher expression of Mcm2 was also reported in PVL cases showing mild and moderate epithelial dysplasia, reflecting a higher proportion of increased G0–G1 cells (Gouvêa et al., 2013; Okoturo et al., 2018).

#### 2.5.4 Clinical and Histological Presentations

**FIGURE 10:**
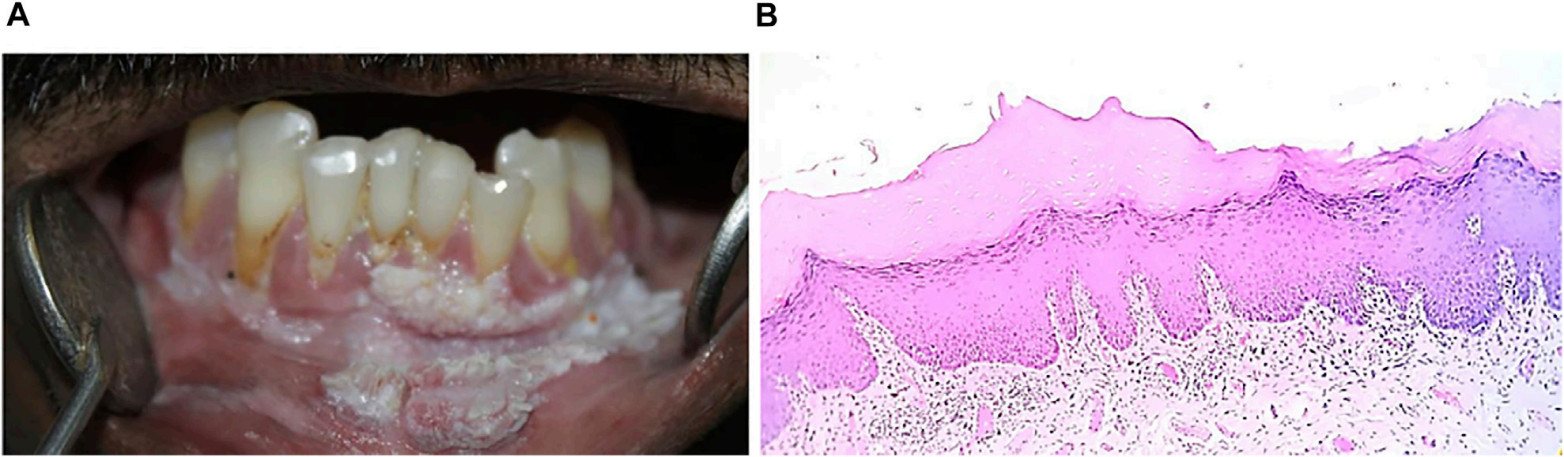
**(A)** Proliferative verrucous leukoplakia show multifocal presence in anterior gingiva, labial vestibule extended to right and left side of gingiva. **(B)** Hyperkeratinized stratified squamous epithelium (4x) with verrucous like projection and inflammatory cell infiltration in lamina propria.

PVL mostly presents as a diffuse hyperkeratosis, a slow-growing, irreversible, persistent disease. Clinically, exophytic wart-like forms of leukoplakia represent PVL and are found to be resistant to nearly all forms of therapy. Clinically, both epithelial hyperkeratosis and verrucous carcinoma or squamous cell carcinoma can be seen in PVL (Greer et al., 1999; Bagan et al., 2010). It is often misdiagnosed by clinicians because of its homogeneously white appearance and its homogeneous (verrucous) texture due to a lack of a precise clinical distinction between verrucous leukoplakia and verrucous carcinoma (Van der Waal, 2015). A histopathological study depends upon the stage of disease, site, and feasibility of biopsy. Lesions show the presence of acanthosis and hyperkeratosis accompanied by lymphocytic infiltrate within lamina propria. Growing lesions show histopathological changes with irregular surfaces and hyperplasia with or without dysplasia (Figure 10) (Morton et al., 2007).

#### 2.5.5 Management and Treatment

PVL is associated with persistent and recurrent growth patterns, and its high propensity to develop into carcinoma demands appropriate treatment. At present, the etiology of PVL is not well understood, and there is a lack of proper management and diagnosis system (Capella et al., 2017). Histological and clinical examinations are considered in PVL diagnosis. Hansen first defined the diagnosis criteria for PVL, later Ghazali et al. (2003) and Gandolfo et al. (2009) also proposed the diagnosis method but, because of the lack of specific histological criteria, none of the methods was able to characterize PVL (Issrani et al., 2013). Common treatment methods include surgery, electrocautery, laser ablation, and cryosurgery (Farah and Savage, 2006). The presence of multiple lesions with early hyperkeratosis to epithelial hyperplasia and atypia progression of hyperplasia, verrucous carcinoma, and eventually to squamous cell carcinoma (SCC) indicate the need for multiple and repeat biopsy and follow-up (Staines and Rogers, 2017).

### 2.6 ORAL Lichenoid Lesions

#### 2.6.1 Definition

OLL, an indistinguishable manifestation of the oral cavity, often presents as an overlapping lesion with OLP. OLL is defined as “Oral lesions with lichenoid features but lacking the typical clinical or histopathological appearances of OLP i.e., may show asymmetry or are reactions to dental restorations or are drug-induced.”

#### 2.6.2 Clinical and Histological Presentations

**FIGURE 11:**
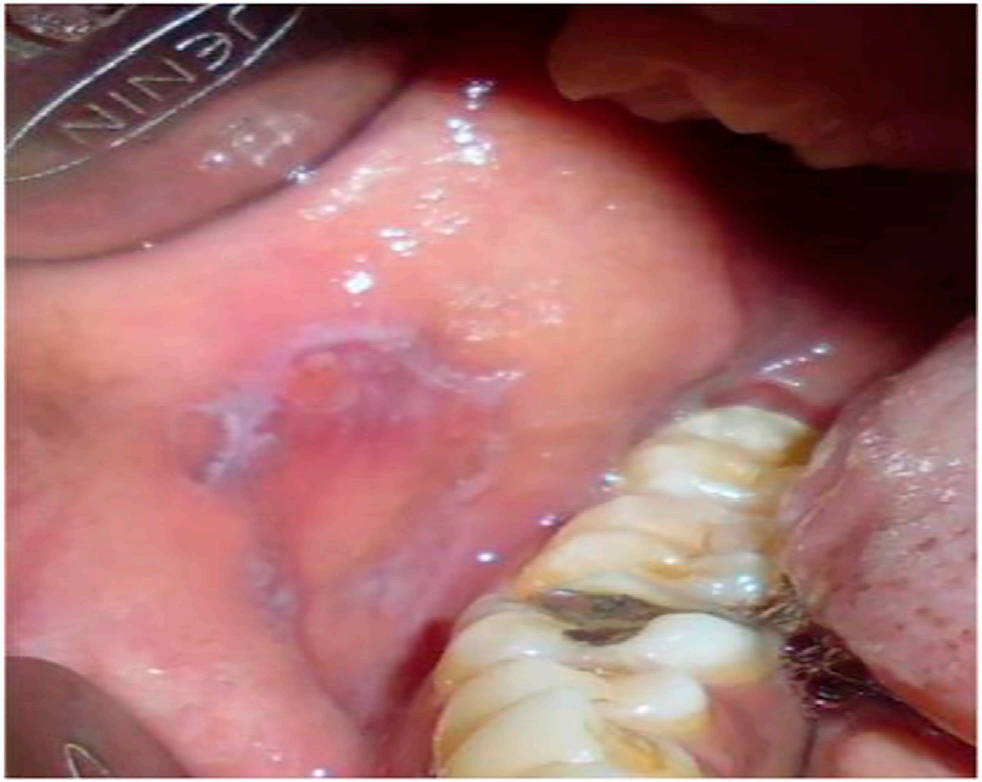
Clinical representation of oral lichenoid lesions.

Typically, OLLs and OLP share similar clinical features and are present as plaques or erosive patches with the presence of Wickham’s striae (Figure 11). However, some distinctive features are also present, like unilateral presentation and morphological changes due to dental amalgam and drugs. Conventionally, OLL can be grouped into different categories based on the mucosal reaction associated with factors: 1) atypical OLP and unilateral lesions, 2) oral contact lesions due to dental reactions, 3) drug lichenoid reactions (DLR), and 4) oral lesions of graft versus host disease. Dental amalgam mostly contains mercury and other corrosive metals like copper and tin, which can be the cause of lichenoid mucosal changes. The gold, palladium, nickel, chrome, and cobalt used in dental restoration can also induce oral lichenoid mucositis. These materials can initiate immune-mediated damage of the basal epithelium and typically appear as an asymmetrical unilateral distribution adjacent to the restorations sites, lateral borders of the tongue, and buccal mucosa (Georgakopoulou and Achtari, 2017). Dental restoration produces type IV hypersensitivity reactions that are mainly related to mercury; only a few cases involving silver, copper, or tin have been reported (Bánóczy et al., 1979; Holmstrup, 1992). Leached mercury salts and other leached metal ions penetrate the epithelial lining, bind to keratinocyte surface proteins, and induce cell-mediated response against basal keratinocytes (Eversole, 1997; Porter et al., 1997; Thornhill, 2001). Drug-mediated oral lichenoid reactions were mostly associated with systemic medications, which include nonsteroidal anti-inflammatory drugs, antihypertensives, oral hypoglycemic agents, beta-blockers, and HIV antiretrovirals (Güneš et al., 2006; Clayton et al., 2010). Immune infiltration is more prominent in DLR and involves the lamina propria. The mechanism by which immune response is initiated is unknown; however, the presence of eosinophil and plasma cells has been seen in immune infiltrate. Langerhans cells and mast cells are also reported to be involved in the pathogenesis of OLL (McCartan and Lamey, 1997). Mast cells release TNF-α, which activates T-cell-dependent ECM modulation. Large numbers of dyskeratotic keratinocytes (colloid or Civatte bodies), perivascular chronic inflammatory cell infiltrate can also be found in OLL (Bariş et al., 2014; Müller, 2017). Histopathological identification of OLL includes more subepithelial infiltrate (eosinophils and plasma cells), perivascular infiltrate, parakeratosis, and colloid bodies. Other features like keratin plugging, atrophy of the rete processes, edema in the lamina propria, and a thick periodic acid-schiff (PAS) deposit in the basement membrane zone were also seen (Solomon et al., 2007; Müller, 2017).

#### 2.6.3 Management and Treatment

The diagnosis of OLL is based on their clinical characteristics and histological findings. However, the sensitivity and specificity of histological diagnosis are very low. In addition, no specific molecular diagnostic markers are present. Epithelial COX-2 and inflammatory COX-2 are overexpressed in OLL in comparison to OLP and are used as a diagnostic marker. Furthermore, COX-1 and COX-2 are found to be overexpressed in oral cancer, where COX-1 is suggested to promote neovascularisation, while the prognostic value of COX-2 has been reported (Pannone et al., 2007). Other diagnostic markers are also reported but they do not provide any definitive distinction between OLL and OLP (Serrano Sánchez et al., 2010; Kamath et al., 2015). Topical corticosteroids are used in the case of symptomatic OLL. Dental-associated lesions involve removal and replacement of dental material to minimize the effect. In cases of DLR, proper evaluation of the risk/benefits of medication is suggested before withdrawal and replacement. After withdrawal, lesions take several months to show remission. Maintenance of proper oral health, removal of plaque, tartar, and films on surfaces of restoration, and use of prophylactic mouth rinsing is suggested (Kamath et al., 2015).

### 2.7 Actinic Cheilitis

#### 2.7.1 Definition

Actinic cheilitis, potentially malignant lesions of the lower lip, is defined as “A disorder that results from Sun damage and affects exposed areas of the lips, most commonly the vermilion border of the lower lip with a variable presentation of atrophic and erosive areas and white plaques” (Lugović-Mihić et al., 2018).

#### 2.7.2 Clinical and Histological Presentations

**FIGURE 12:**
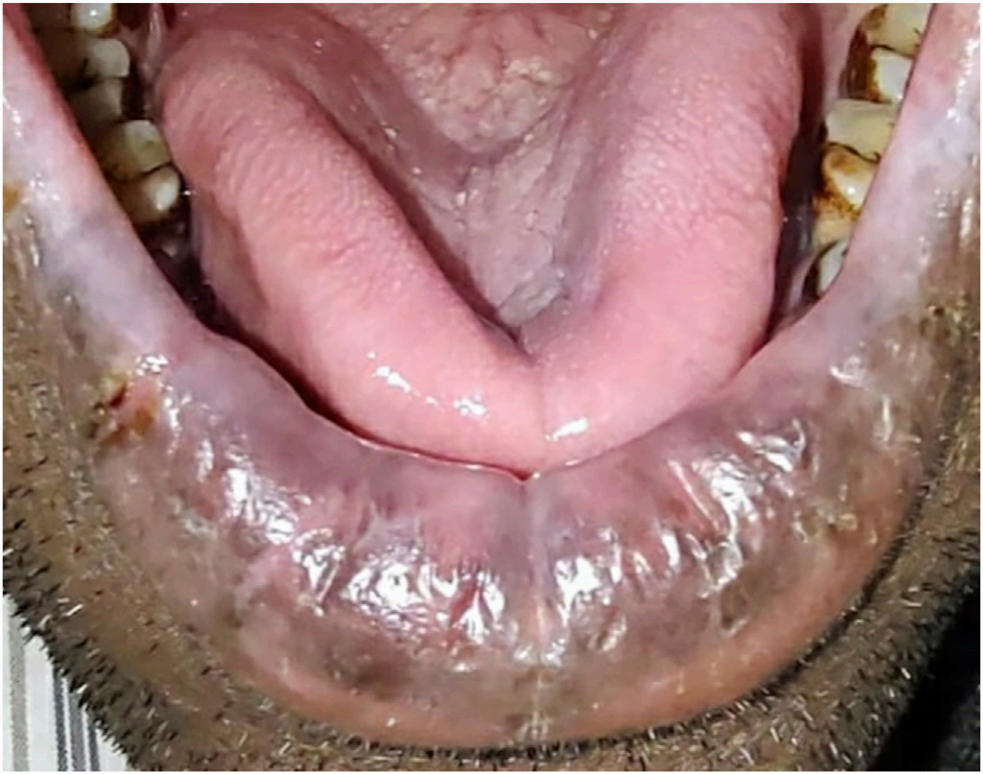
Actinic cheilitis a typically presentation at lower lip.

Long-term ultraviolet exposure is the main etiological factor, and outdoor workers are thus primarily affected, particularly fair-skinned people (Maruccia et al., 2012; Piñera-Marques et al., 2010). It is a chronic condition persistent throughout the year, and the overall prevalence is 2.08%. Hyperkeratosis, dryness, and atrophy are identifiable clinical features (Figure 12). White lip plaque found in AC is a late symptom present after atrophy. Hyperkeratotic plaques frequently progress from early homogeneity to an opaque non‐homogeneous plaque. Other clinical features include scaly sites, swelling, erythema, ulceration, indistinct vermilion border, transverse fissures, white plaque, crusting, blotchiness, and tissue pallor (Cavalcante et al., 2008; Poitevin et al., 2017). Histological features include the presence of dysplasia, solar elastosis, inflammation, vasodilatation, hyperplasia, hyperkeratosis, and epithelial atrophy. MT is generally accounted for by recurrent ulceration with delayed wound healing, red and white spots with loss of the vermilion border, persistent crusting and flaking, atrophic appearance with opaque white thickening, and focal induration or nodule formation (Picascia and Robinson, 1987; Savage et al., 2010). Regulatory T cells (Tregs) are involved in immunosuppression in oral cancer. Expression of Foxp3+Tregs cells inhibits T-cell proliferation, generating an immunosuppressive microenvironment with high levels of suppressor cytokines (IL-10 and TGF-β) (Gasparoto et al., 2014).

#### 2.7.3 Management and Treatment

Management and treatment are mainly based on demographic, clinical, and histopathologic findings (Markopoulos et al., 2004). Both surgical and non‐surgical treatment methods have been employed which include cryotherapy, electrosurgery, topical retinoids, 5‐fluorouracil cream, imiquimod cream, photodynamic therapy, carbon dioxide laser ablation, and surgical vermilionectomy (Savage et al., 2010).

### 2.8 Palatal Lesions in Reverse Smokers

#### 2.8.1 Definition

Palatal lesions in reverse smokers are defined as “White and/or red patches affecting the hard palate in reverse smokers, frequently stained with nicotine” (Warnakulasuriya et al., 2020a). Reverse smoking is a unique form of smoking where the burning end of the cigarette is kept inside the mouth. It is more common in women with poor socioeconomic backgrounds. The habit of reverse smoking is seen in parts of India, the Caribbean Islands, Colombia, Panama, Venezuela, Jamaica, Sardinia, and the Philippines (Warnakulasuriya, 2018).

#### 2.8.2 Clinical and Histological Presentation

**FIGURE 13:**
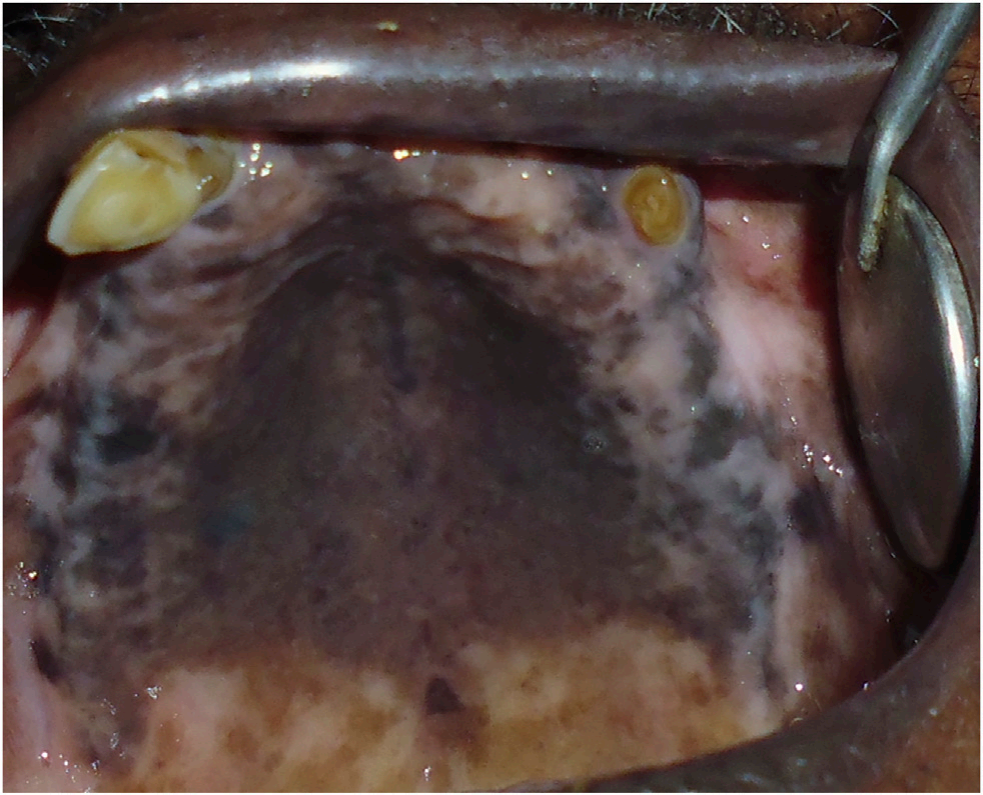
Clinical presentation of palatal lesions in reverse smokers.

Clinical symptoms of the oral mucosa in people with a reverse smoking habit have been documented to differ from those in conventional smokers. The palate and tongue are the affected areas (Figure 13). Lesions associated with this habit are present as palatal keratosis, excrescences, leukoplakia, and ulcerations to frank malignancy (Bharath et al., 2015a). Furthermore, abnormalities like mucosal thickness, fissuring, pigmentation, nodularity, erythema, and ulceration were seen in people associated with this habit (Bharath et al., 2015b). Palatal hyperpigmentation is due to increased melanin synthesis by melanocytes causing a greyish black appearance. The production of melanin is a protective reaction against heat and acts as an antioxidant against toxic products produced during tobacco combustion in reverse smoking. In some cases, palatal mucosal consisting of splotchy areas of depigmentation surrounded by hyperpigmentation was also reported. These observations were reported in people with heavy drinking and smoking habit. Furthermore, depigmentation was linked to a lack of melanin synthesis, and hyperchromasia of the basal epithelium was discovered (Bharath et al., 2015b).

#### 2.8.3 Management and Treatment

Ramulu et al. proposed a grading system for reverse smokers based on premalignant abnormalities in the palate, which included no palatal changes (Grade 0), mild (Grade 1), moderate (Grade 2), severe (Grade 3) form, and “palatal carcinoma” (Grade 4) (Ramulu et al., 1973; Ramesh et al., 2014). Palliative treatment, such as quitting the smoking habit and use of antioxidants, is a common practice for oral lesions. Curcumin formulation was shown to heal the palatal alterations linked with reverse smoking habit; however, a detailed study is needed (Vijayalaxmi et al., 2012).

### 2.9 ORAL Lupus Erythematosus

#### 2.9.1 Definition

Oral lupus erythematosus is defined as “An autoimmune connective tissue disease which may affect the lip and oral cavity, where it presents as an erythematous area surrounded by whitish striae, frequently with a ‘target’ configuration” (Banerjee and Chatterjee, 2015; Warnakulasuriya et al., 2020a).

#### 2.9.2 Clinical and Histological Presentation

Lupus erythematosus is a chronic immunological disorder that mainly consists of three types: 1) systemic, 2) drug-induced, and 3) discoid. All of the types can be seen in oral mucosa. Oral manifestations of systemic lupus erythematosus (SLE) show ulceration, honeycomb plaque, raised keratotic plaque, nonspecific erythema, purpura, petechiae, and a cheilitis-like morphology (Chi et al., 2010). Oral lesion manifestation occurs in 25% of SLE patients. The most common manifestation is oral ulcerations with a prevalence of 7%–41% (Harvey et al., 1954). Oral manifestations of discoid lupus erythematosus are called “Oral discoid lesions.” Typically, it affects the sun-exposed areas of the face and neck and may present as butterfly rash across the nasal bridge. Buccal mucosa, gingiva, labial mucosa, and the vermillion border were the most common sites for oral discoid lupus erythematosus (Ranginwala et al., 2012a). Clinical features are similar to OLP and are often confused during diagnosis (Ranginwala et al., 2012b). Subepithelial immunoglobulin and complement deposition (the lupus band) are present, and they help to distinguish between OLP and OLE (Warnakulasuriya, 2018). The presence of white papules, the central erythema, a border zone of irradiating white striae, and peripheral telangiectasia are all common clinical features of oral discoid lesions. Histological features define the presence of hyperortho and/or parakeratosis (hyperparakeratosis), liquefaction degeneration of the basal layer, and perivascular lymphocyte infiltration. Other characteristics include the presence of keratotic plugs, epithelial collagen degradation, a decrease in stratum granulosum thickness, and blood vessel thickening (Ranginwala et al., 2012a).

#### 2.9.3 Management and Treatment

Antimalarial drug hydroxychloroquine is the first choice in treating LE. This treatment carries a minor risk of developing retinopathy, which is reversible once the drug is stopped. There is also the possibility of oral mucosal melanin pigmentation. Oral manifestations of LE, on the other hand, do not always resolve after systemic treatment. Furthermore, because Candida infection is common in oral LE lesions, antimycotic therapy should be used. This type of therapy can be used in conjunction with systemic immunosuppressive therapy. Following a few days of antimycotic therapy (which should be continued for 2–4 weeks), topical steroids similar to those used to treat oral lichen lesions should be used. Routine oral examination has also been suggested as one of the preventive measures in LE patients. Oral ulcers are common in LE and patients refractory to therapy should be referred for a histological examination to rule out malignancy (Brennan et al., 2005; Benli et al., 2021).

### 2.10 Dyskeratosis Congenita

#### 2.10.1 Definition

Dyskeratosis Congenita is described as “A rare cancer-prone inherited bone marrow failure syndrome caused by aberrant telomere biology. It is characterized clinically by the presence of the diagnostic triad of dysplastic nails, lacy reticular skin pigmentation and oral leukoplakia” (Ballew and Savage, 2013).

#### 2.10.2 Clinical and Histological Presentation

Oral leukoplakia is frequently present in DC and is found in 65%–80% of patients. The inheritance of DC can be X-linked (Zinsser-Cole-Engleman syndrome), autosomal dominant (dyskeratosis congenita, Scoggins type), or autosomal recessive. In some cases, sporadic and mutational changes can also be associated with DC (Gitto et al., 2020). A total of six genes related to the maintenance of telomerase structure and function have been identified to be mutated, and these are DKC1, TERC, TERT, TINF2, NOLA2, and NOLA3 (Savage et al., 2009). DC was diagnosed when two of the three triad symptoms of dysplastic nails, lacey reticular pigmentation of the upper chest and/or neck, and oral leukoplakia were present. Oral symptoms include the appearance of white, thicker patches on the mouth’s mucous membranes (oral leukoplakia). These patches appear gradually, usually in the second, third, or fourth decade of life (Gitto et al., 2020). Oral and dental abnormalities such as hypodontia, short blunted roots, hypocalcification, thin enamel, gingival recession, gingival inflammation with edema, gingival bleeding, alveolar bone loss, periodontitis, extensive caries, smooth atrophic tongue mucosa, leukoplakia, and lichen planus have been reported in DC (Elad, Aljitawi, Zadik).

#### 2.10.3 Management and Treatment

DC signs and symptoms can develop at any age, and they usually get worse as they get older. The diagnosis of DC involves extensive evaluation of telomere length. Methods like terminal restriction fragment (TRF) measurement on Southern blots, fluorescence *in situ* hybridization (FISH) with immunostaining, quantitative PCR, single telomere length analysis, and flow-cytometry with FISH (flow-FISH) are widely used. The medical management of DC is complex, and immunosuppressive therapy does not work for most patients. It is recommended to use oxymetholone in low doses because it can cause liver enzyme abnormalities (Velazquez and Alter, 2004; Savage et al., 2009).

### 2.11 Oral Graft versus Host Disease

#### 2.11.1 Definition

GVHD is a condition caused by antigen incompatibility between the donor and recipient’s HLA systems. It is the most common and devastating complication of hematopoietic stem cell transplantation (HSCT), and it is considered the major cause of late mortality unrelated to the underlying malignancy. GVHD is an autoimmune and alloimmune disorder that affects many organs and tissues (Margaix-Muñoz et al., 2015).

#### 2.11.2 Clinical and Histological Presentation

The clinical and histopathological attributes of GVHD are similar to those of OLP (Warnakulasuriya et al., 2020a). GVHD is mainly associated with hematopoietic stem cell or bone marrow transplants, which are widely used for the treatment of different benign and malignant hematological diseases. GVHD is the most common and serious complication of HSCT. Clinical symptoms involve mucosal hyperkeratotic, erythema, inflammation, atrophy, pseudomembranous ulcerations, fibrosis, salivary gland dysfunction, and taste disorders similar to autoimmune disorders of LP, lupus, systemic sclerosis, and Sjogren syndrome. Clinically, it can be acute or chronic (a/cGVHD). The skin, gastrointestinal tract, and liver are the most commonly affected organs in aGVHD. The oral cavity is one of the most commonly affected regions in cGVHD with a frequency of 45%–83%. The clinical presentation of oral cGVHD involves mucosal inflammation, atrophy, lichenoid-hyperkeratotic changes (striae, plaques, papules, and patches), pseudomembranous ulcerations, mucoceles, and perioral fibrosis. Other characterization includes vasculitis-like structure, telangiectatic, sclerotic changes in the perioral tissues may result in the decreased oral opening. Patients usually reported oral pain, sensitivity to normally tolerated items, xerostomia, and occasionally taste dysfunction (Elad, Aljitawi, Zadik; Schubert and Correa, 2008; Mays et al., 2013). The known risk factors for developing cGVHD include an old age recipient, female donor to male recipient, MHC mismatch, total body irradiation (TBI) conditioning, unrelated donors, a peripheral blood stem cell source, donor lymphocyte infusion, and prior acute GVHD (Flowers et al., 2011). Histologically, the presence of dyskeratotic epithelial cells, apoptosis, and inflammatory infiltrate consisting of CD3^+^ and CD68 ^+^ T cells beneath the epithelial basal lamina are seen. cGVHD is distinguished by fibrosis caused by collagen deposition and atrophy (Imanguli et al., 2006; Margaix-Muñoz et al., 2015).

#### 2.11.3 Management and Treatment

There is no specific drug therapy available for GVHD. Commonly prescribed drugs include corticosteroids with or without calcineurin inhibitors. Prednisone is recommended in severe cases. Different complications such as secondary infections, osteoporosis, hypertension, hyperglycemia, renal failure, and hyperlipidemia are often associated with cGVHD treatment. When corticosteroid treatment fails, second- and third-line treatment options are considered. Drugs such as sirolimus, everolimus, pentostatin, rituximab, and imatinib are recommended for second-line treatment. Third-line therapy consists of mofetil mycophenolate, methotrexate, and corticosteroid pulses. While the use of hydroxychloroquine, clofazimine, cyclophosphamide, alemtuzumab, anti-TNFα (infliximab, etanercept), thoracoabdominal irradiation, thalidomide, alefacept, daclizumab/basiliximab, retinoids, azathioprine, and mesenchymal stem cells are less common in cGVHD (Dignan et al., 2012).

## 3 OPMDs Screening and Management

Management of OPMDs is focused on cessation of risk factors and surgical excision of lesions with moderate and severe epithelial dysplasia. Non-surgical approaches involve the use of systemic and topical medications. Early screening of the lesions is important to ensure timely diagnosis or follow-up in case of confusing lesions to minimize the malignant transformation. Many screening techniques have been developed and are widely used by clinicians. Some of the screening aids include 1) vital staining with toluidine blue (TB) and Lugol’s iodine (LI), 2) devices based on autofluorescence, 3) devices based on chemiluminescence, 4) narrow-band imaging (NBI), 5) high-frequency ultrasounds, 6) optical coherence tomography (OCT), 7) *in vivo* confocal microscopy, and 8) biomarker assessment (from saliva, serum, or exfoliated cells) (Tables 2, 3) (Lingen et al., 2008; Patton et al., 2008; Romano et al., 2021). Histological diagnosis has also emerged as a powerful technique in evaluating the type of oral lesion and determining the malignant potential. Despite efforts to improve the management of OPMDs, many cases are still detected so late that a cure is very difficult, and interventions are of limited efficacy. Therefore, detection of OPMDs at an early stage, especially in high-risk groups is of utmost importance to prevent its further progression to malignancy. It is precisely important to risk-stratify the patients based on numerous factors, like the size, clinical appearance, and histology of the lesion to provide appropriate counseling and screening for higher-risk individuals. Further, patients with a habit of tobacco chewing, areca nut consumption, alcoholism, smoking, and drug abuse are at high risk. Thus, it is very important to educate the public regarding the dangers of these factors (Sugerman and Savage, 2002).

**TABLE 2:** Non-invasive OPMDs screening technique.

Techniques	Procedures
Vital staining with toluidine blue (TB) and Lugol’s iodine (LI)	TB selectively stains neoplastic/dysplastic lesions rich in nucleic acids (high DNA and RNA content) and appears royal blue (TB positive) while normal tissue does not take up dye (TB negative)
	LI stains healthy buccal mucosa tissue. Where iodine reacts with glycogen (iodine–starch reaction) and normal mucosa, the locations appear to be brown or orange in color. Enhanced glycolysis in cancer cells does not promote the reaction
Devices based on autofluorescence	Oral tissue irradiated with any blue light at the wavelength of 400–460 nm excites endogenous fluorophores such as keratin, collagen, elastin, and NADH to emit green light (500–520 nm). The degree of emission of green light decrease and appears as a black spot as dysplasia progression to cancer due to an increase in hemoglobin, porphyrins, and melanin, which tends to absorb the blue incident light, reducing the emission of green light
Devices based on chemiluminescence	This consists of a disposable capsule with two compartments containing acetylsalicylic acid and an inner glass vial containing hydrogen peroxide. Breaking of the vial triggers the reaction of the chemicals contained in the two compartments. Consequently, a bluish-white light (430–580 nm) is produced; it lasts for 10 min and facilitates the identification of hyperkeratotic areas
	Another chemiluminescence device contains the combination of chemiluminescence and toluidine blue (TB). Before taking the image, 1% acetic acid solution is used to rinse the mouth for 1 minute, to desiccate oral tissues, followed by an oral examination with 430–580 nm wavelength light. The altered epithelial cells appear as “aceto-white” lesions, whereas normal cells appear blue
Narrow-Band Imaging (NBI)/virtual chromoendoscopy with magnification (VCM)	This is a novel endoscopic technique where optical filters are used to narrow the light bandwidth to enhance the visualization of the mucosal surface and microvasculature. Generally, blue (415 nm) and green lights (540 nm) are used as they are strongly absorbed by hemoglobin
Optical coherence tomography (OCT)	High-resolution cross-sectional imaging is done by OCT. It is based on low-coherence interferometry, when a ray of light (an electromagnetic wave) reflects and diffuses on the tissues in different ways, resulting in the echo time delay and intensity of backscattered or back-reflected light from internal tissues
High-Frequency Ultrasound (US)	Piezoelectric crystals are used to emit sound waves, and their echoes are capable of producing images of anatomical structures
*In Vivo* Confocal Microscopy	Perform virtual biopsy of the tissues in their living context, offering real-time cytological and histological details. Laser light at specific wavelengths is used to stimulate endogenous fluorophores resulting in the emission of fluorescent or refracted light from the living tissue

**TABLE 3:** List of identified genetic alteration at chromosome level and tissue marker present in OPMDs.

OPMD Type	Chromosome Altered	Tissue Marker
Leukoplakia	Deletion - 3p14, 4q, 8p, 9p21, 11q, 17p	p53, PD-L1
	Allelic imbalance- 3p21, 8p21-23, 9p21, 13q14.2, 17p13.1, 18q21.1	
	CNV- 3p, 8p, 9p, 11q, 13q, 18q, 7p	
Oral lichen planus	Genetic alteration - 3p, 9p, 17p	CD4^+^, CD8^+^ T cell, TLR-2, p53, TNF-α, IL-6, COX-2, and CD34
	Monosomy of chromosome 9	
Oral submucous fibrosis	LOH- 1, 3, 4, 6, 7, 9, 10, 11, 12, 13, 15, 18, 19, 20	TGF-β
Erythroplakia	Polysomy of chromosomes 7 and 17	p53
	LOH or allelic gain at 9p, and 3p	
Proliferative verrucous leukoplakia	Allelic loss at 9p21 (INFα, D9S1748, and D9S171)	p53, Mcm2
Oral cancer	CNV- 3, 5, 7, 8, 9, 11	p53

## 4 Conclusion

Despite advances in cancer therapeutics, the mortality rate of oral cancer is high. An understanding of the conditions leading to oral cancer and their prevention can result in better outcomes. The OPMDs are premalignant conditions that can transform into oral cancer in many cases, if not detected and treated early. Therefore, an understanding of their identification, pathogenesis, and prevention is of great importance.

The worldwide prevalence rate of OPMDs is approximately 5%. Moreover, this estimated data varies significantly from country of study. They are responsible for a significant number of oral cancer cases worldwide. In epidemiological studies assessing the risk of OPMDs in India, it has been found that 80% of oral cancers were preceded by OPMDs.

The present work provides insight into the risk factors, characteristics, and pathogenesis of OPMDs. It also emphasizes the importance of early diagnosis and prevention of OPMDs. A better understanding of the nuances of the OPMDs may help in treating physicians to stratify patients as per their risk and accordingly treatment strategy can be decided.

The scientific research on OPMDs is still in infancy and research efforts are required to develop better diagnostic, prognostic, and therapeutic strategies. It is hoped that better diagnostic and prognostic abilities will be immensely useful for the clinical management of OSCC.
